# The gut microbiota in multiple sclerosis varies with disease activity

**DOI:** 10.1186/s13073-022-01148-1

**Published:** 2023-01-05

**Authors:** Florence Thirion, Finn Sellebjerg, Yong Fan, Liwei Lyu, Tue H. Hansen, Nicolas Pons, Florence Levenez, Benoit Quinquis, Evelina Stankevic, Helle B. Søndergaard, Thomas M. Dantoft, Casper S. Poulsen, Sofia K. Forslund, Henrik Vestergaard, Torben Hansen, Susanne Brix, Annette Oturai, Per Soelberg Sørensen, Stanislav D. Ehrlich, Oluf Pedersen

**Affiliations:** 1 Université Paris-Saclay, INRAE, MGP, 78350 Jouy-en-Josas, France; 2 Danish Multiple Sclerosis Center, Department of Neurology, Copenhagen University Hospital – Rigshospitalet, 2600 Glostrup, Denmark; 3 Department of Clinical Medicine, University of Copenhagen, 2200 Copenhagen, Denmark; 4 Novo Nordisk Foundation Center for Basic Metabolic Research, Faculty of Health and Medical Science, University of Copenhagen, 2200 Copenhagen, Denmark; 5 Center for Clinical Research and Prevention, Bispebjerg and Frederiksberg University Hospital, 2400 Frederiksberg, Denmark; 6 Experimental and Clinical Research Center, A Cooperation of Charité–Universitätsmedizin and the Max-Delbrück Center, 10117 Berlin, Germany; 7 Max Delbrück Center for Molecular Medicine (MDC), 13125 Berlin, Germany; 8 Charité–Universitätsmedizin Berlin, 10117 Berlin, Germany; 9 DZHK (German Centre for Cardiovascular Research), Partner Site Berlin, 10785 Berlin, Germany; 10 Structural and Computational Biology Unit, European Molecular Biology Laboratory, 69117 Heidelberg, Germany; 11 Department of Medicine, Rønne Hospital, 3700 Bornholm, Denmark; 12 Department of Biotechnology and Biomedicine, Technical University of Denmark, 2800 Kongens Lyngby, Denmark; 13 Department of Department of Clinical and Movement Neurosciences, UCL Queen Square Institute of Neurology, London, WC1N 3RX UK; 14 Center for Clinical Metabolic Research, Herlev-Gentofte University Hospital, Hellerup, 2900 Copenhagen, Denmark

**Keywords:** Multiple sclerosis, Shotgun sequencing, Gut microbiota, *Gordonibacter urolithinfaciens*, *Faecalibacterium prausnitzii*

## Abstract

**Background:**

Multiple sclerosis is a chronic immune-mediated disease of the brain and spinal cord resulting in physical and cognitive impairment in young adults. It is hypothesized that a disrupted bacterial and viral gut microbiota is a part of the pathogenesis mediating disease impact through an altered gut microbiota-brain axis. The aim of this study is to explore the characteristics of gut microbiota in multiple sclerosis and to associate it with disease variables, as the etiology of the disease remains only partially known.

**Methods:**

Here, in a case-control setting involving 148 Danish cases with multiple sclerosis and 148 matched healthy control subjects, we performed shotgun sequencing of fecal microbial DNA and associated bacterial and viral microbiota findings with plasma cytokines, blood cell gene expression profiles, and disease activity.

**Results:**

We found 61 bacterial species that were differentially abundant when comparing all multiple sclerosis cases with healthy controls, among which 31 species were enriched in cases. A cluster of inflammation markers composed of blood leukocytes, CRP, and blood cell gene expression of *IL17A* and *IL6* was positively associated with a cluster of multiple sclerosis-related species. Bacterial species that were more abundant in cases with disease-active treatment-naïve multiple sclerosis were positively linked to a group of plasma cytokines including IL-22, IL-17A, IFN-β, IL-33, and TNF-α. The bacterial species richness of treatment-naïve multiple sclerosis cases was associated with number of relapses over a follow-up period of 2 years. However, in non-disease-active cases, we identified two bacterial species, *Faecalibacterium prausnitzii* and *Gordonibacter urolithinfaciens*, whose absolute abundance was enriched. These bacteria are known to produce anti-inflammatory metabolites including butyrate and urolithin. In addition, cases with multiple sclerosis had a higher viral species diversity and a higher abundance of *Caudovirales* bacteriophages*.*

**Conclusions:**

Considerable aberrations are present in the gut microbiota of patients with multiple sclerosis that are directly associated with blood biomarkers of inflammation, and in treatment-naïve cases bacterial richness is positively associated with disease activity. Yet, the finding of two symbiotic bacterial species in non-disease-active cases that produce favorable immune-modulating compounds provides a rationale for testing these bacteria as adjunct therapeutics in future clinical trials.

**Supplementary Information:**

The online version contains supplementary material available at 10.1186/s13073-022-01148-1.

## Background

Multiple sclerosis is a chronic immune-mediated disease of the brain and spinal cord, resulting in physical and cognitive impairment in young adults [1, 2]. Demyelination and axonal injury, the histopathology hallmarks of multiple sclerosis, are thought to arise from an immune-mediated attack on myelinated axons and the myelin sheath, involving CD4+ T cells, cytotoxic CD8+ T cells, B cells, and macrophages [3]. Most cases (about 85%) suffer from relapsing-remitting multiple sclerosis (RRMS) having clinical relapses with worsening of existing or new neurological symptoms and disease activity in the form of white matter brain lesions that can be visualized on magnetic resonance imaging (MRI) scans [4]. There is still no cure for multiple sclerosis and the long-term outcome is unpredictable, but disease-modifying therapies affecting pathogenic immune reactions are available for the RRMS subtype [5]. Treatments such as interferon beta, glatiramer acetate, teriflunomide, and dimethyl- and diroximel-fumarate provide a modest decrease in disease activity, whereas treatments such as sphingosine-l-phosphate receptor modulators (fingolimod, ozanimod, ponesimod), natalizumab, anti-CD20 monoclonal antibodies (rituximab, ocrelizumab, ofatumumab), cladribine, and alemtuzumab are more efficacious but some of these can be more burdensome for the patients due to a higher risk of severe side effects [5, 6].

The etiology of multiple sclerosis is complex and incompletely understood. More than 200 genetic variants associated with multiple sclerosis have been identified in genome-wide association studies while the heritability for multiple sclerosis is estimated to be only 19% [7]. Several environmental risk factors, including smoking, Epstein-Barr virus infection (infectious mononucleosis), obesity in childhood and adolescence, and vitamin D deficiency have been identified, but the overall contribution of these risk factors to absolute disease risk may be rather limited, suggesting the existence of additional environmental risk factors [8].

The human gastrointestinal tract is a habitat for a large number of commensal and mutualistic microbes collectively known as the gut microbiota, and the collective genome of microbiota, known as the gut microbiome, contains about an order of magnitude more genes than the human genome [9]. The gut microbiota is hypothesized to be implicated in the pathogenesis of neurological diseases [10] and since disturbances of the gut microbiota might lead to a pro-inflammatory activation of the immune system, it has been suggested that an altered gut microbiota might be an additional disease mechanism in multiple sclerosis [11–13].

Experimental autoimmune encephalomyelitis (EAE) is an accepted mouse model of multiple sclerosis and initial studies in this animal model showed that the gut microbiota was essential for activation of pathogenic, myelin-reactive CD4+ T cells while germ-free mice were protected against disease development [14]. In human twin studies, transplantation of fecal samples from twins suffering from multiple sclerosis to mice led to a higher rate of spontaneous EAE than did fecal samples from healthy co-twins [15]. Interestingly, transplantation of fecal samples from healthy co-twins were associated with higher production of interleukin 10 (IL-10) that is a cytokine with multiple effects in immunoregulation and anti-inflammatory processes [16]. Blocking IL-10 in recipients of fecal samples from healthy co-twins increased the incidence of EAE [15]. Other studies have indicated that treatment with a human commensal—*Prevotella histocola*—is as efficacious as the multiple sclerosis therapies interferon beta and glatiramer acetate in ameliorating disease in the EAE model [17, 18].

It has been suggested that gut dysbiosis might lead to an altered balance between short-chain fatty acids (SCFAs), which have immunoregulatory, including anti-inflammatory effects, and long-chain fatty acids with pro-inflammatory and disease-promoting effects in EAE, but there is no strong evidence that this is also the case in multiple sclerosis [19, 20]. In pediatric multiple sclerosis, both individual and clusters of various gut microbes were associated with longitudinal disease activity, and the known functions and metagenomics predictions of these microbes suggest an important role of butyrate and amino acid biosynthesis pathways [21]. A recent study reported that individuals with multiple sclerosis had lower serum concentrations of propionic acid and that treatment with propionic acid inhibited the development of EAE and promoted the expansion of regulatory T cells by an effect mediated by changes in the gut microbiota [22]. Low serum concentrations of propionic acid or other SCFAs have, however, not been found in all studies of multiple sclerosis [23]. Similarly, a recent systematic review and other recent original studies failed to find evidence of a consistent pattern of changes in gut microbiota in multiple sclerosis [24, 25].

The objective of our study was to map the intestinal microbiota applying shotgun-sequencing-based gut metagenome analyses in a prospectively collected cohort of recently diagnosed Danish multiple sclerosis cases and matched healthy controls (HC), and relate bacterial and viral gut microbiota features to blood biomarkers of inflammation, targeted blood cell gene expression, and clinical course of multiple sclerosis. Previous studies of the intestinal microbiota biomarkers of multiple sclerosis and other neurological disorders have largely failed to account for effects of various treatment regimens and inter-individual variability of bacterial cell load of stool sample [20, 26, 27]. These shortcomings are accounted for in the present study. Moreover, we monitored disease activity in multiple sclerosis cases over a period of 2 years and related the clinical course of patients with intestinal microbiota features at baseline.

## Methods

### Study population

Multiple sclerosis cases were recruited from the outpatient clinic of the Danish Multiple Sclerosis Center, Department of Neurology, Rigshospitalet University Hospital, Copenhagen, in the period April 2013 to June 2014. When seen in the outpatient clinic, patients were invited to participate in the study. Inclusion criteria were RRMS or clinically isolated syndrome (CIS), Danish ethnicity, and age 18–60 years; exclusion criteria were other autoimmune or known cancer disease or other conditions (gut disorders, metabolic syndrome, psychiatric and mental disorders) that might affect the gut microbiota. Multiple sclerosis cases were evaluated at a baseline visit where all patients had a neurological examination by the same neurologist and delivered fasting blood samples and a fecal sample. Clinical data on the patients including age, sex, disease duration, expanded disability status scale (EDSS), and multiple sclerosis severity score (MSSS) were registered. All had a baseline cerebral MRI scan, and a follow-up scan 2 years later to evaluate radiological disease activity by number of new white matter lesions in the brain [4].

A relapse was defined according to the 2017 McDonald diagnostic criteria [28]. Clinical disease activity at baseline was evaluated by number of relapses 1 year prior to the baseline visit and was dichotomized as clinically not active (CNA, no relapse) or clinically active (CA, one relapse or more). Number of relapses, worsening in EDSS, new white matter lesions on MRI, and NEDA-3 (no evidence of disease activity, i.e., no relapses, no new/enlarging white matter MRI lesions and stable EDSS) were monitored during the follow-up period of 2 years.

HC subjects who reported no acute or chronic disorders were selected among individuals who were age- and sex-matched with multiple sclerosis cases from (1) the population-based DanFunD cohort (*n* = 88), recruited among Danish citizens as described by Dantoft et al. [29] and (2) individuals phenotyped at Novo Nordisk Foundation Center for Basic Metabolic Research, University of Copenhagen (CBMR; *n* = 60) (unpublished). The CMBR cohort was recruited from urban areas in Denmark by advertisement in local newspapers, social media, and other online resources from November 2013 to November 2014.

Originally, 152 multiple sclerosis patients were included in the study, but four individuals were excluded from further analyses due to sample mix-up, leading to a final sample size of 148 Danish cases and 148 age- and sex-matched Danish HC subjects.

Blood was drawn in the morning after an overnight fast from a cubital vein into an EDTA tube, centrifuged to separate plasma and cells, and immediately stored at −80°C until analysis. Collected plasma samples were further used for metabolic markers and cytokine measurement (all individuals).

For untreated cases only, whole blood was collected in PAXgene tubes with the PAXgene miRNA Blood kit (PreAnalytiX, Qiagen) at the same time as plasma collection and was further subjected to microarray gene expression measurement.

Stools were collected according to International Human Microbiome Standards (IHMS) guidelines (SOP 03 V1) in kits by multiple sclerosis cases and HC at home and immediately stored at −20 °C until they were transported on dry ice and frozen 4–24 h later at −80°C in plastic tubes at the biobanks of Novo Nordisk Foundation Center for Metabolic Research or Glostrup Hospital. Stools were further subjected to shotgun sequencing (all individuals), bacterial cell counting (all individuals), and fecal water estimation (cases only).

Written informed consent was obtained from all study participants. The study protocol involving multiple sclerosis cases and HC was approved by the Ethical Committees of the Capital Region of Denmark (Protocol no.: H-4-2012-176). The DanFunD study (H-3-2012-015) and the CBMR study (H-3-2012-145) were also approved by the Ethical Committees of the Capital Region of Denmark.

### Measurement of plasma cytokines

Plasma cytokines were determined by high-sensitivity immunoassays based on electrochemiluminescence (Meso Scale Discovery). Samples were pre-diluted two times and analyzed according to the manufacturer’s instructions, except for sample incubation time, which was performed overnight at 4°C on a shaker to improve assay sensitivity. Each plate contained a biomarker-specific internal standard in duplicate as well as two blank wells. Samples were analyzed in duplicates and read on a Sector Imager 2400A (Meso Scale Discovery, Gaithersburg, MD, USA). Concentrations were calculated using 8-point standard curves. The lower levels of detection as well as the percentage of detectable samples are detailed in Additional file 1: Table S1. Undetectable values in the low end were set at half the minimum value of the given cytokine; all high-end values were detectable due to the high dynamic range of the assays.

### Microarray gene expression in whole blood

RNA was extracted from whole blood collected in PAXgene tubes with the PAXgene miRNA Blood kit (PreAnalytiX, Qiagen) in the fasting state. RNA integrity and concentration were analyzed on a 2100 Bioanalyzer (Agilent Technologies, DK). A minimum of 450 ng of total RNA with mean RIN values of 8.9 was used as input. RNA was amplified and labelled using the WT PLUS reagent kit (Thermo Fisher Scientific, Carlsbad, CA, USA). The labelled samples were hybridized to the Human Gene 2.0 ST array (Affymetrix, Santa Clara, CA, USA). The arrays were washed and stained with phycoerytrin-conjugated streptavidin using the Affymetrix Fluidics Station® 450, and the arrays were scanned in the Affymetrix GeneArray® 3000 scanner to generate fluorescent images, as described in the Affymetrix GeneChip® protocol. Cell intensity files (CEL files) were generated in the GeneChip® Command Console® Software (AGCC) (Affymetrix, USA). The microarray data were modelled using the RMA (Robust Multichip Average) approach, followed by mean one step probe set summarization giving each gene a single expression value, all done using the software package Partek Genomics Suite 6.

### Bacterial cell counting

For bacterial cell counting, 0.08-0.12 g of frozen (−80 °C) fecal samples were diluted 15 times in pH 7.2 DPBS (Sigma-Aldrich), mechanically homogenized using tissue lyser (40 min, 12.5 agitations per second; QIAGEN) and fixed with 2% paraformaldehyde (10 min, RT; Biotum). Then the samples were diluted 120 times in filtered staining buffer (1 mM EDTA, 0.01% Tween20, pH 7.2 DPBS, 1% BSA; (Sigma-Aldrich)). To minimize clumps, the samples were filtered through a cell strainer (pore size 5 μm; pluriSelect), pre-wet in the staining buffer. Next, the bacterial cell suspension was stained with SYBR Green I (1:200,000 (Fisher Scientific), in DMSO (Sigma-Aldrich)) and incubated in the dark for 30 min. For accurate determination of bacterial cell count, a known concentration of 123count eBeads (Invitrogen) was added to the samples prior to the analysis. Measurements were performed using a BD Fortessa LSRII flow cytometer (BD Biosciences), and data were acquired using BD FACSDiVaTM software. A threshold value of 200 was applied on the FITC (530/30 nm) channel. Fluorescence intensity at green (530/30 nm, FITC), blue (450/50 nm, Pacific Blue), yellow (575/26 nm, PE), and red (695/40 nm, PerCP-Cy5-5) fluorescence channels as well as forward- and side-scattered (FSC and SSC) light intensities were collected. Measurements were performed at a pre-set flow rate of 0.5 μL/s. Data were processed in R using flowcore package in R Studio. Fixed gating strategy separated the microbial fluorescent events from the fecal sample background (Additional file 2: Fig. S1). Individual bacterial cell counts are given in Additional file 1: Table S2.

### Fecal water content estimation

For estimating fecal water content in stools from multiple sclerosis cases, frozen feces samples were weighed before and after freeze-drying. Freeze-drying included a primary drying performed at 0.1hPa and 23°C for 17h and a secondary drying at 0.05 hPa and 23°C for 3h (CoolSafe touch 15L, LaboGene, Lilleroed, Denmark). Individual data for fecal water content is given in Additional file 1: Table S2.

### Stool sampling, DNA extraction, and shotgun sequencing

DNA extraction from aliquot of fecal samples was performed following IHMS SOP P7 V2 [30, 31]. DNA was quantitated using Qubit Fluorometric Quantitation (Thermo Fisher Scientific, Waltham, US) and qualified using DNA size profiling on a Fragment Analyzer (Agilent Technologies, Santa Clara, US). Three micrograms of high molecular weight DNA (>10 kbp) was used to build the library. Shearing of DNA into fragments of approximately 150 bp was performed using an ultrasonicator (Covaris, Woburn, US) and DNA fragment library construction was performed using the Ion Plus Fragment Library and Ion Xpress Barcode Adapters Kits (Thermo Fisher Scientific, Waltham, US). Purified and amplified DNA fragment libraries were sequenced using the Ion Proton Sequencer (Thermo Fisher Scientific, Waltham, US), with a minimum of 20 million high-quality reads of 150 bp (in average) generated per library.

### Gene count table

To construct a gene count table, METEOR software was used [32]: first, reads were filtered for low quality by AlienTrimmer [33]. Reads that aligned human genome (identity > 95%) were also discarded. Remaining reads were mapped onto the Integrated Gut Catalogue 2 (IGC2) [34], comprising 10.4 million of genes, using Bowtie2 [35]. The unique mapped reads (reads mapped to a unique gene in the catalogue) were attributed to their corresponding genes. Then, the shared reads (reads that mapped with the same alignment score to multiple genes in the catalogue) were attributed according to the ratio of their unique mapping counts of the captured genes. The resulting count table was further processed using the R package *MetaOMineR* v1.31 [36]. It was downsized at 12 million mapped reads to take into account differences in sequencing depth and in mapping rate across samples. Then the downsized matrix was normalized for gene length and transformed into a frequency matrix (FPKM normalization). Gene count was computed as the number of genes present (abundance strictly positive) in the frequency matrix.

### Profiling and annotation of MetaGenomics Species (MGS)

The IGC2 was previously organized into 1990 MetaGenomics Species (MGS) using MSPminer [37, 38]. Relative abundance of an MGS was computed as the mean abundance of its 100 “marker” genes (that is, the genes that correlate the most altogether). If less than 10% of “marker” genes were seen in a sample, the abundance of the MGS was set to 0. For a given sample, cell count index was computed as the cell count of this sample normalized by the mean cell count over all measured samples. Missing values were imputed by 1. MGS relative abundance were further corrected by this index to take into account difference in bacterial cell count between samples. In this way, we estimated absolute abundance of bacterial species.

Abundances at higher taxonomical ranks were computed as the sum of the MGS that belong to a given taxa. MGS count was assessed as the number of MGS present in a sample (that is, whose abundance is strictly positive).

### Predicted functional modules of gut bacteriome

Three databases were used to predict gene functions: Kyoto Encyclopedia of Genes and Genomes (KEGG) [39], eggNOG [40], and TIGRFAM [41]. Genes from the IGC2 catalogue were mapped with diamond [42] onto KEGG orthologs (KO) from the KEGG database (version 8.9). Each gene was assigned to the best-ranked KEGG orthologs (KO) among hits with e-value < 10e−05 and bit score > 60. The same procedure was used with eggNOG (version 3.0). The gene catalogue was searched against TIGRFAM profiles (version 15.0) using HMMER 3.2.1 [43]. Then we assessed presence of KEGG modules, gut metabolic modules (GMMs) [44] and gut-brain modules (GBMs) [45] in an MGS. A functional module consists in an ensemble of KOs (or NOGs, or TIGRFAMs). Since MGS are pangenomes, their genes are divided into “core” genes (which are present in all samples harboring the MGS) or “accessory” (which might be absent from a sample even if the MGS is detected). Thus, we first considered a functional module to be present in an MGS if at least 90% of its components were present in the “core” genes of the MGS. Then we re-affined this assumption sample by sample, by adding to the “core” genes the accessory genes detected in a given sample. Finally, we measured the potential of a module in a sample by summing abundances of all MGS found to carry this module in this sample.

### Analyses of viral gut microbiota

The viral gut microbiota was analyzed using MiCoP [46], as this method is optimized to call viruses directly from the bulk metagenomics sequencing reads. As a reference dataset, MiCoP draws upon the NCBI’s RefSeq Viral database [47]. We identified a total of 150 viral species with prevalence of > 10% and relative abundance of > 0.01% for 296 (148 multiple sclerosis cases versus 148 HC) individuals included in the dataset.

### Statistical analysis applied in analyses of bacterial and viral gut microbiota

All statistical analysis were performed with R v3.6.0 [48]. Contrasts in MGS or functional modules abundances were performed using Mann-Whitney test if two groups and Kruskal-Wallis if more than two groups. Correlations between variables (either metagenomics variables or clinical variables) were performed using Spearman’s correlations. All *p*-values were corrected for multiple testing with the Benjamini-Hochberg method. Unless stated otherwise, a corrected *p*-value (*q*-value) is assessed as significant when under the threshold of 0.1. Effect size was computed as the Cliff’s Delta (CD) using the package *effsize* v0.7.4 [49].

Bray-Curtis dissimilarity was computed on the log-10 transformed MGS table with the package *vegan* v2.5.7 [50]. Principal coordinates analysis (PCoA) was performed on the Bray-Curtis dissimilarity with the package *ade4* v1.7.16 [51]. Bray-Curtis dissimilarity variance between groups was then analyzed by PERMANOVA with the function *adonis* from the package *vegan*.

Covariates deconfounding was performed on each metagenomics feature with the R package *metadeconfoundR* v0.1.5 [52, 53]. Covariates included status (multiple sclerosis cases or HC, CA, or CNA, respectively), BMI, age, sex, fecal water content, and medication. When a metagenomics feature is significantly associated with at least two covariates, these covariates can be strictly deconfounded, ambiguously deconfounded, or confounded [52].

For viral gut microbiota analysis only, differences in abundance were detected using Microbiome Multivariable Association with Linear Models (MaAslin2) [54] and corrected for multiple testing by Benjamini-Hochberg method. Unless stated otherwise, a corrected *p*-value (*q*-value) was assessed as significant when under the threshold of 0.1.

## Results

### Cohort characteristics

Stools from 148 cases with multiple sclerosis and 148 sex- and age-matched healthy controls (HC) were sampled (Table 1). All study participants were white Danish individuals. There were more current smokers among cases than among HC (27% and 10%, respectively, *P* = 3.5e−06, chi-squared test). The majority (86%) of cases had RRMS, according to the 2017 McDonald criteria [28], while 14% had a CIS with only one relapse and not fulfilling the 2017 McDonald criteria for RRMS (Table 1). Cases had various medication profiles: 36% had no treatment, while 23 and 41% had first-line and second-line treatment, respectively (Table 1, Additional file 1: Table S3). We measured a series of cytokines in fasting plasma of all study participants and found after accounting for covariates (age, sex, BMI, smoking, and drug treatment) that the plasma concentration of chemokine ligand 2 (CCL2) was higher in cases, whereas plasma concentrations of transforming growth factor beta (TGF-β) and interkeukin-1 beta (IL-1β) were lower (Additional file 1: Table S1, Additional file 2: Fig. S2).

**Table 1:** Demographic characteristics of the patients with multiple sclerosis and healthy controls

**Variable**	**Patients with multiple sclerosis**	**Healthy controls**	***P***^a^
N	148	148	-
Age (years), mean ± SD	36 ± 8.4	36 ± 8.4	0.48
Sex (Female/Male), n(%)	98(66)/50(34)	98(66)/50(34)	1
BMI (kg/m²), mean ± SD	24 ± 4.3	23 ± 3.4	0.078
Smoking (Never/Previous/Current), n(%)	50(34)/56(38)/40(27)	90(61)/41(28)/15(10)	3.5e-06

*SD* Standard deviation

^a^*P*-values associated either with Wilcoxon test (quantitative variable) or Chi-squared test (qualitative variable) are displayed

### Contrasted bacterial taxa and predicted functional modules of the bacterial gut microbiota in multiple sclerosis cases and healthy controls

Both gut bacterial gene richness and metagenomic species (MGS, hereafter termed species) richness were similar in cases and HC (Additional file 2: Fig. S3A-B). Global bacterial microbiota composition (beta diversity) was different between cases and HC (*P* < 0.001, PERMANOVA, Additional file 2: Fig. S3C). Removal of current and former smokers from analyses did not change the result (Additional file 2: Fig. S4). Intriguingly, when computing pairwise PERMANOVA between treatment-based subgroups of cases and HC, we found that each subgroup was significantly different from HC apart from the treatment-naïve cases (*P* < 0.05, Additional file 1: Table S4, Additional file 2: Fig. S5A), suggesting that global differences in beta diversity might be due to treatment of multiple sclerosis. Regarding species richness, only the subgroup of patients treated with Gilenya (*n* = 17) was different from HC (*P* = 0.036). In particular, species richness was similar between HC and treatment-naïve patients (*P* = 0.48, Additional file 2: Fig. S5B).

**Fig. 1:**
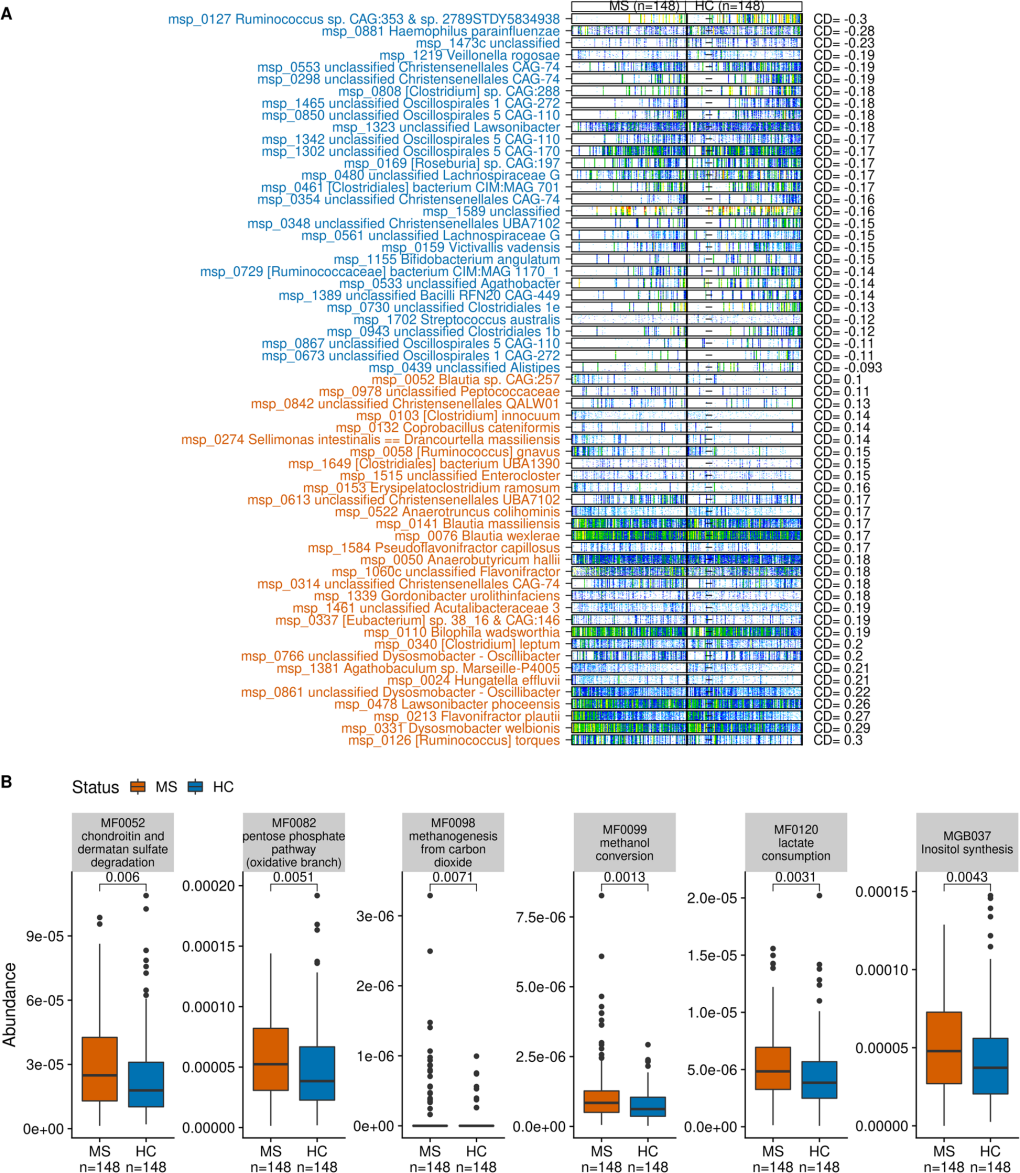
Contrasting bacterial species (metagenomics species (MGS)) and functional modules. **A** Barcode illustration of contrasted bacterial species after deconfounding for covariates (age, sex, BMI, smoking, and drug treatment). The 50 “tracer” genes are in rows, abundance is indicated by color gradient (white, not detected; red, most abundant); individuals, ordered by status (cases or HC) and by increasing species richness, are in columns. **B** Boxplots of contrasted bacterial modules (gut metabolic module (GMM) and gut-brain module (GBM)) after deconfounding for the same covariates. *P*-values associated with Wilcoxon test are displayed. MS = multiple sclerosis patients, HC = healthy controls

We found that abundance of 61 species (10% of all examined species) was different between HC and cases after accounting for covariates. Covariates included age, sex, BMI, smoking, and drug treatment. Half of them were enriched in multiple sclerosis cases (referred to as MS-related species, *n* = 31), and half of them were depleted (HC-related species, *n* = 30, Fig. 1A). The multiple sclerosis-related species included *Ruminococcus torques*, *Dysosmobacter welbionis*, *Flavonifractor plautii*, *Lawsonibacter phoceensis*, *Hungatella effluvia*, *Bilophila wadsworthia*, *Gordonibacter urolithinfaciens*, *Anaerobutyricum hallii*, *Pseudoflavonifractor capillosus*, *Blautia wexlerae*, *Blautia massiliensis*, *Anaerotruncus colihominis*, *Erysipelatoclostridium ramosum*, *Ruminococcus gnavus*, *Sellimonas intestinalis*, *Coprobacillus cateniformis*, and *Clostridium innocuum*. The HC-related species included *Haemophilus parainfluenzae*, *Veillonella rogosae*, *Victivallis vadensis*, *Bifidobacterium angulatum*, and *Streptococcus australis*. Most multiple sclerosis-related species (65%) were inversely correlated with species richness, while most HC-related species (87%) were positively correlated with richness when considering the total cohort (*n* = 296 individuals), or specific subgroups (HC, cases or treatment-naïve cases, respectively) (Additional file 1: Table S5, Additional file 2: Fig. S6).

We analyzed the predicted functional modules of the bacteriome issued from three databases: gut-brain modules (GBM), gut metabolic modules (GMM), and KEGG modules. Comparing cases and HC, we found one, five, and zero contrasted modules, respectively, all more abundant in cases (Fig. 1B). The only contrasted GBM was the inositol synthesis pathway. The five contrasted GMM were chondroitin sulfate and dermatan sulfate degradation, pentose phosphate pathway (oxidative branch), methanogenesis from carbon dioxide, methanol conversion, and lactate consumption.

### Contrasted bacterial species correlate with plasma inflammation markers and blood cell gene expression

**Fig. 2:**
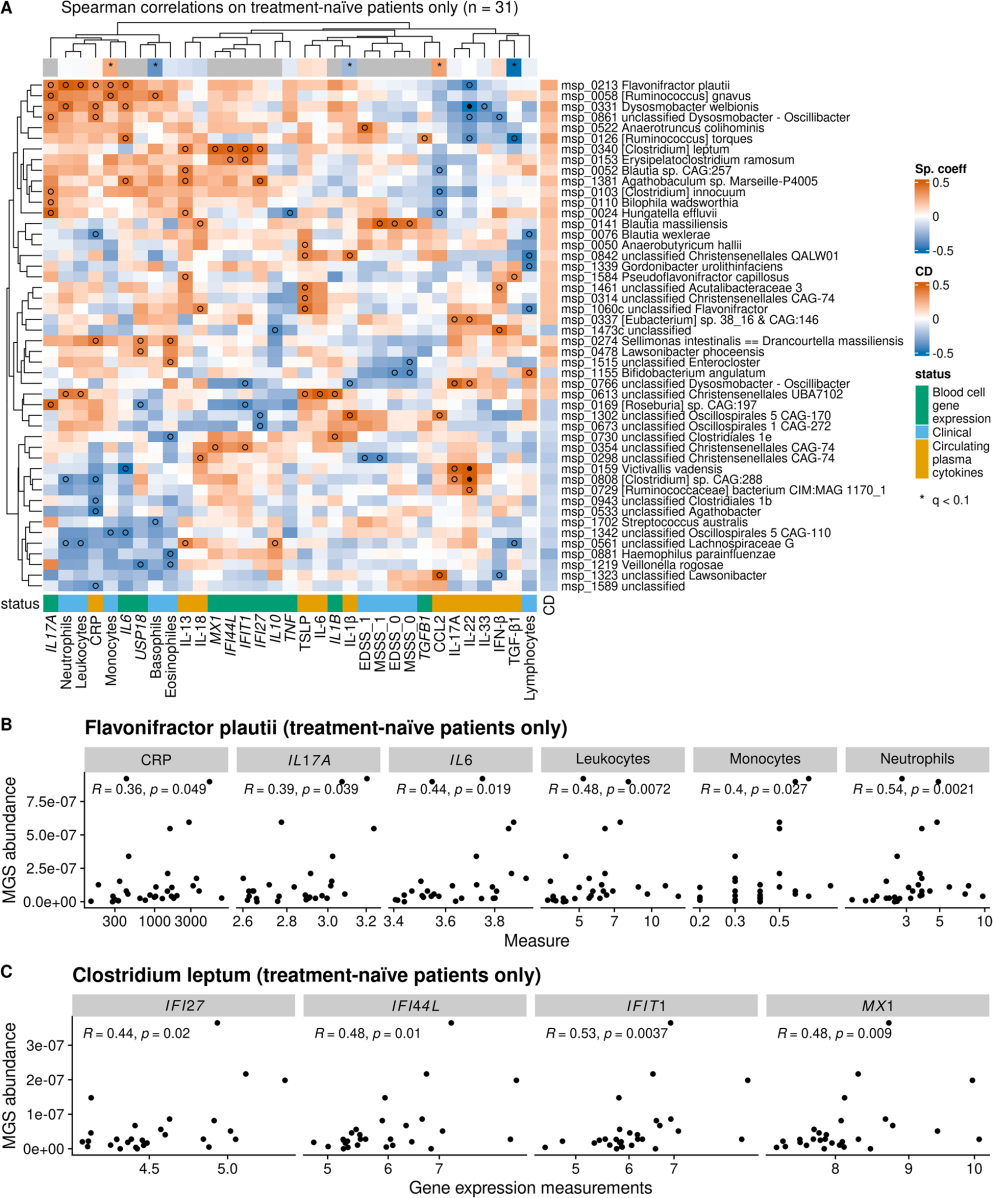
Associations of contrasted bacterial species (metagenomics species (MGS)) with inflammatory markers. **A** Spearman’s correlations between contrasted bacterial species and fasting circulating inflammatory markers in the subgroup of treatment-naïve patients only. Only features with at least one *p*-value under 0.05 are displayed. Black dots denote correlations with *FDR* ≤ 0.1, while empty circles indicate correlation with *P* ≤ 0.05. The right side bars indicate the Cliff’s Delta (CD, effect size) of the feature in the cases/HC contrast (red: more abundant in cases; blue: more abundant in HC). **B** Relationships between abundance of *Flavonifractor plautii* and a group of fasting circulating inflammation markers. **C** Relationships between abundance of *Clostridium leptum* and expression of selected blood leukocyte genes. Spearman’s correlation coefficients along with the associated *p*-values are displayed. CD = Cliff’s Delta; MGS = metagenomics species. MS = multiple sclerosis patients, HC = healthy controls, EDSS = expanded disability status scale; MSSS = multiple sclerosis severity score (0: at baseline; 1: after 2-year follow-up)

Since markers of inflammation intendedly are influenced by multiple sclerosis treatment, we correlated contrasted bacterial species with clinical variables, plasma cytokine concentrations, and blood cell gene expression in treatment-naïve cases only (Fig. 2A). A cluster of inflammation markers (composed of blood leukocytes, CRP, and blood cell gene expression of *IL17A* and *IL6*) was positively associated with a cluster of MS-related species (among which *Flavonifractor plautii* had the highest number of significant correlations, *P* < 0.05, Fig. 2B) while inversely associated with a cluster of HC-related species. This pattern of associations, as well as the specific correlations of *F. plautii* with markers of inflammation was confirmed in HC (Additional file 2: Fig. S7A-B). Moreover, MS-related species *Clostridium leptum* correlated directly with expression of four type 1 IFN-induced blood cell genes: *MX1*, *IFIT1*, *IFI44L*, and *IFI27* (Fig. 2C).

### Bacterial species richness of treatment-naïve multiple sclerosis cases associate with number of relapses

**Fig. 3:**
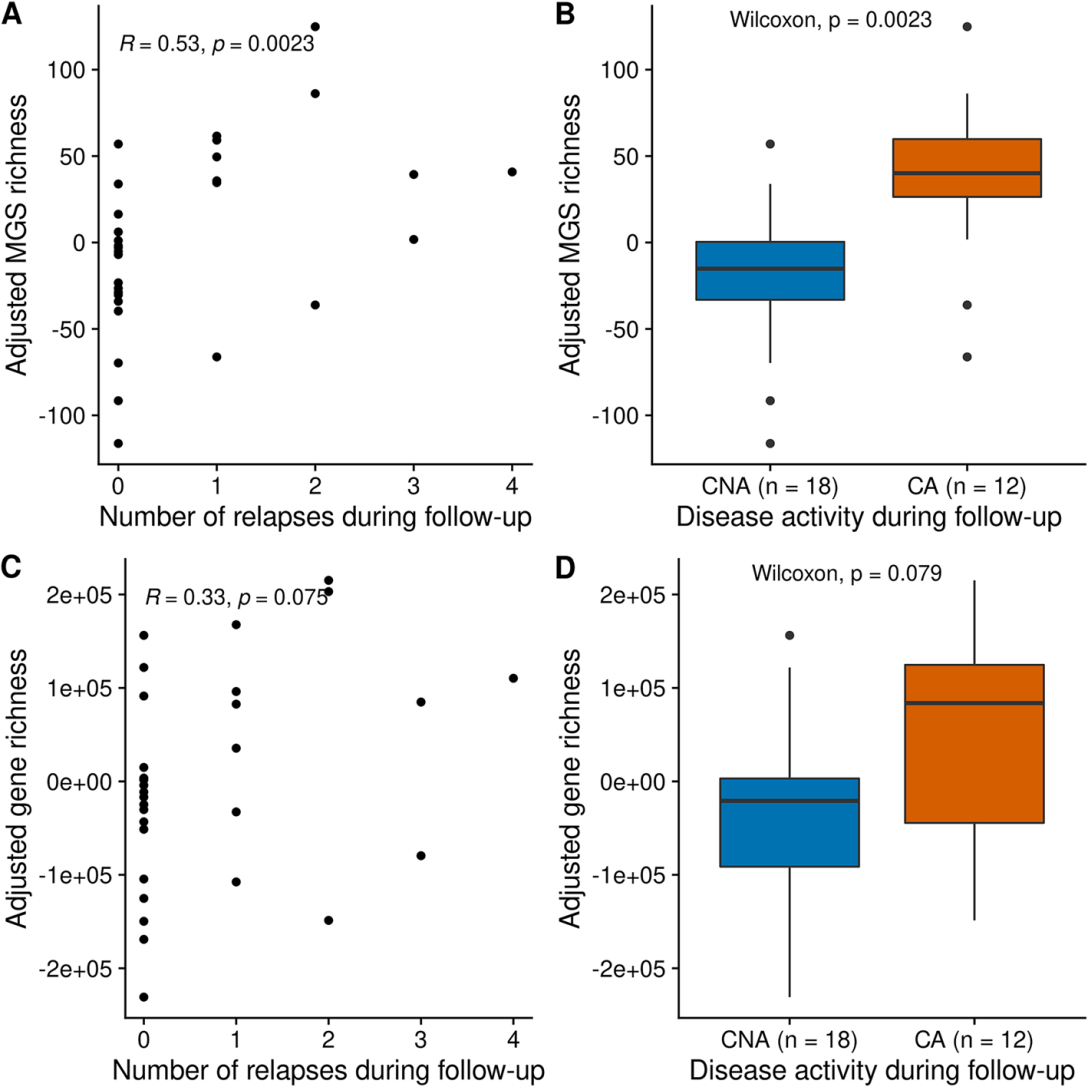
Species (metagenomics species (MGS)) richness and disease activity in treatment-naïve patients. **A–C** Relationships between **A** species richness or **C** gene richness adjusted for covariates (age, sex, BMI, smoking status, and fecal water content) and number of relapses during follow-up, in treatment-naïve patients only. Spearman’s correlation coefficients along with the associated *p*-values are displayed. **B–D** Distribution of **B** adjusted species richness or **D** adjusted gene richness, according to disease activity in treatment-naïve patients. *P*-values associated with Wilcoxon tests are displayed. CNA = clinically not active; CA = clinically active; MS = multiple sclerosis patients, MGS = metagenomics species

In treatment-naïve cases (*n* = 31), species richness adjusted for age, sex, BMI, fecal water content (a proxy of constipation), and smoking status was unexpectedly correlated with the number of relapses over 2 years of follow-up (*rho* = 0.53, *P* = 0.002, Spearman’s correlation, Fig. 3A). Consistently, the group of clinically active (CA, i.e., at least one relapse during the 2 years of follow-up; *n* = 12) among treatment-naïve cases was significantly richer in bacterial species than the group of clinically not active cases (CNA; i.e., no relapse during the 2 years of follow-up; *n* = 19) (*P* = 0.023, Wilcoxon test, Fig. 3B). Bacterial gene richness showed the same trend (Fig. 3C,D). There was no such relationship in the other treatment-based subgroups, including the group of cases that was formerly treated (*n* = 23, *rho* = 0.2, *P* = 0.36, Spearman’s correlation, Additional file 2: Fig. S8). Adjusted species richness was not associated with duration between baseline and latest relapse before baseline or first relapse after baseline (Additional file 2: Fig. S9A-B-C).

**Fig. 4:**
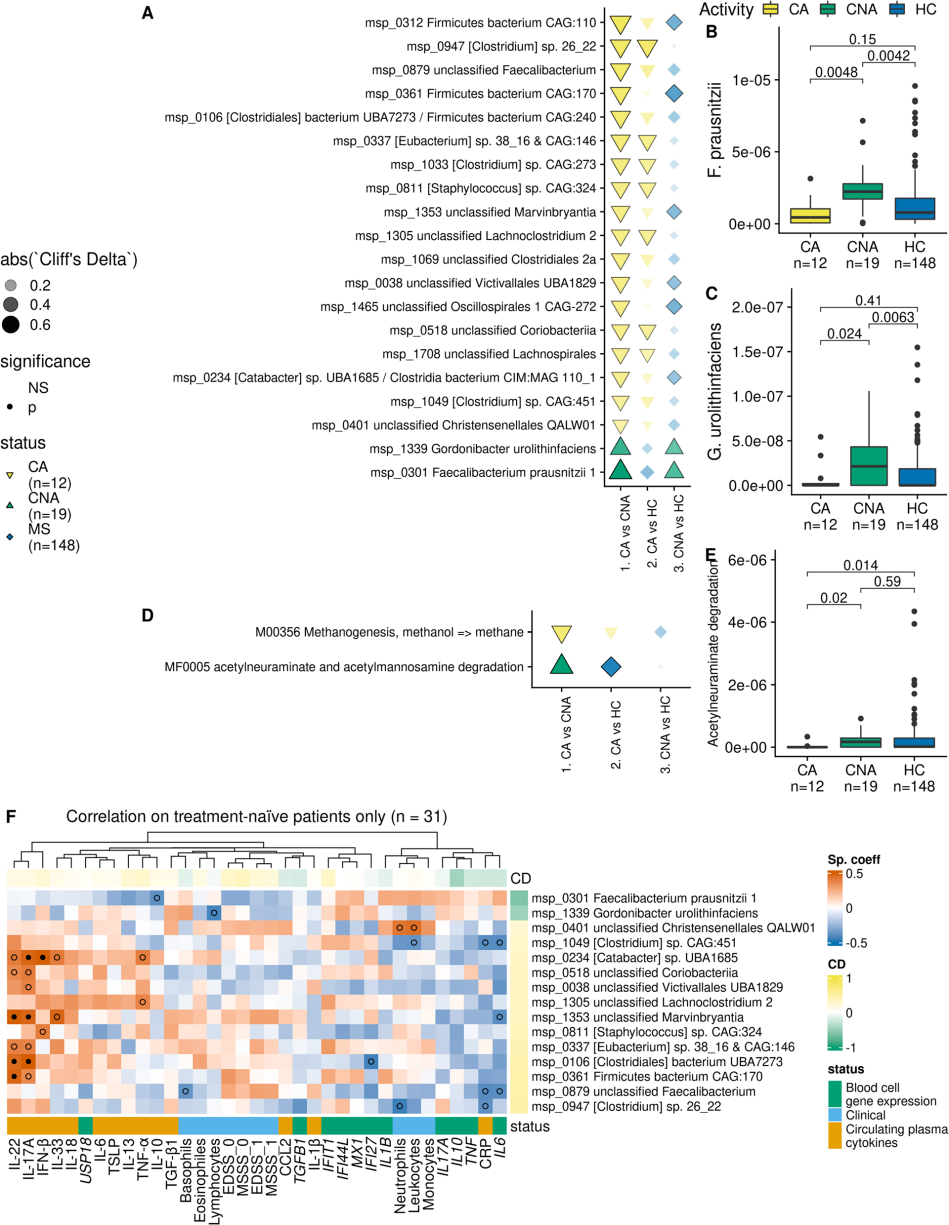
Bacterial species (metagenomics species (MGS)) and bacteriome modules related to MS activity. **A** Bacterial species and **D** predicted bacteriome functional modules that are contrasted between CA and CNA treatment-naïve patients (after deconfounding for age, sex, BMI, smoking status, and fecal water content). Along is their effect size (Cliff’s Delta) in the contrasts (1) CA vs CNA, (2) CA vs HC, (3) CNA vs HC. **B**,**C**,**E** Distribution of bacterial species or bacteriome functional modules that are more abundant in CNA patients. *P*-values associated with Wilcoxon tests are displayed. **F** Correlation between contrasted bacterial species and fasting circulating inflammation markers in treatment-naïve patients. Only bacterial species with at least one *p*-value under 0.05 are displayed. Black dots denote correlations with *FDR* ≤ 0.1, while empty circles indicate correlation with *P* ≤ 0.05. The right side bars indicate the Cliff’s Delta (CD, effect size) of the feature in the CA/CNA contrast (green: more abundant in CNA; yellow: more abundant in CA). CD = Cliff’s Delta; CA = clinically active; CNA = clinically not active; HC = healthy controls; MS = multiple sclerosis patients; NS = non-significant, EDSS = expanded disability status scale; MSSS = multiple sclerosis severity score (0: at baseline; 1: after 2-year follow-up)

CA and CNA cases from the treatment-naïve group had similar phenotypic profile, except for BMI (Table 2). When contrasting abundance of bacterial species between CA and CNA cases, two species were more abundant in CNA after deconfounding for covariates (*P* ≤ 0.05): *Faecalibacterium prausnitzii* and *Gordonibacter urolithinfaciens*. The same two species were also more abundant in CNA cases when comparing these to HC but displayed no difference between CA and HC (Fig. 4A–C). Apart from *G. urolithinfaciens*, only two species from the HC/MS contrast were found significantly different between CA and CNA (an *Anaerobutyricum* and an unclassified Oscillospirales). In particular, *F. plautii* showed no difference between CA and CNA (Additional file 2: Fig. S10).

**Table 2:** Demographic and clinical characteristics of the clinically active and clinically non-active treatment-naïve patients

	**CA**	**CNA**	***P***^a^
N	12	19	-
Age (years), mean ± SD	34 ± 9.4	39 ± 8	0.16
Sex (Female/Male), n(%)	8(67)/4(33)	11(58)/8(42)	0.91
BMI (kg/m²), mean ± SD	22 ± 2.3	24 ± 2.7	0.029
Smoking (Never/Previous/Current), n(%)	5(42)/3(25)/4(33)	4(21)/8(42)/7(37)	0.43
Fecal water content (%), mean ± SD	67 ± 12	71 ± 10	0.16
EDSS (baseline), mean ± SD	2.1 ± 1	1.5 ± 1.4	0.17
MSSS (baseline), mean ± SD	5 ± 2.1	3.6 ± 2.8	0.092
EDSS (follow-up), mean ± SD	2.2 ± 1.5	1.8 ± 1.8	0.36
MSSS (follow-up), mean ± SD	4 ± 2.1	3.3 ± 3	0.41
Number of relapses during follow-up, mean ± SD	1.8 ± 1	0 ± 0	1.3e-07

*CA* Clinically active patient, *CNA* Clinically non-active patient, *SD* Standard deviation

^a^*P*-values associated either with Wilcoxon test (quantitative variable) or Chi-squared test (qualitative variable) are displayed

At the bacterial functional level, acetylneuraminate and acetylmannosamine degradation potentials were increased in CNA, whereas methanogenesis (methanol => methane) was increased in CA (Fig. 4D,E). Interestingly, other methanogenesis-related features were found either ambiguously deconfounded (*Methanobrevibacter* (genus), Methanobacteriaceae (family)), or confounded by fecal water content (coenzyme M biosynthesis, F420 biosynthesis, CO_2_ => methane) (Additional file 1: Table S6). Consistently, abundance of the genus *Methanobrevibacter* and fecal water content were inversely correlated, considering all MS patients or only those carrying the genus *Methanobrevibacter* (*rho* = −0.24, *P* = 0.003, *n* = 146, and *rho* = −0.29, *P* = 0.027, *n* = 57, respectively) (Additional file 2: Fig. S11).

Bacterial species more abundant in CA treatment-naïve cases were positively correlated to a group of plasma cytokines including IL-22, IL-17A, IFN-β, IL-33, and TNF-α, while inversely correlated to CRP, and the blood cell gene expression of *IL6* (Fig. 4F). Species more abundant in CNA treatment-naïve cases showed the opposite pattern, though generally not significantly. More specifically, *F. prausnitzii* negatively correlated with the cytokine IL-10 (*rho =* −0.39, *P* = 0.03, *n* = 31) while *G. urolithinfaciens* inversely correlated with lymphocytes counts (*rho* = −0.43, *P* = 0.02, *n* = 31). Of note, only blood cell gene expression of *IL10* displayed a significant difference between CA and CNA (*P* = 0.048) after adjusting for covariates. All bacterial species more abundant in CA were positively correlated with bacterial species richness (*rho* > 0) in the different sub-cohorts (treatment-naïve cases only, all cases, HC or all cohort, respectively). Consistently, abundance of *G. urolithinfaciens* was inversely correlated with species richness in the same sub-cohorts (−0.3 < *rho* < −0.25) whereas abundance of *F. prausnitzii* was inversely correlated with species richness in treatment-naïve cases (*rho* = −0.18), and tended to be positively correlated with species richness in other sub-cohorts (0.06 < *rho* < 0.09, Additional file 2: Fig. S12).

Interestingly, in HC, the abundance of *F. prausnitzii* was positively correlated to total blood leukocyte and neutrophil counts, and to the plasma cytokines CCL2 and IL-33 (*P* < 0.05, Additional file 2: Fig. S13).

Considering all CNA and CA cases (*n* = 100 and *n* = 48, respectively) yielded similar results at the bacterial species level: the set of species significantly enriched in CNA still included *F. prausnitzii* and *G. urolithinfaciens* and was further enriched with *Anaerostipes hadrus*, *Gemmiger formicilis*, and *Roseburia inulinovorans*. On the other hand, absolute abundance of *Methanobrevibacter smithii* and *Victivallis vadensis* was enriched in CA (Additional file 2: Fig. S14). At predicted bacterial functional levels, results were different, with propionate degradation increased in CNA, while coenzyme M biosynthesis and lysine biosynthesis were enriched in CA (Additional file 2: Fig. S15).

### Alterations of the viral gut microbiota in multiple sclerosis

**Fig. 5:**
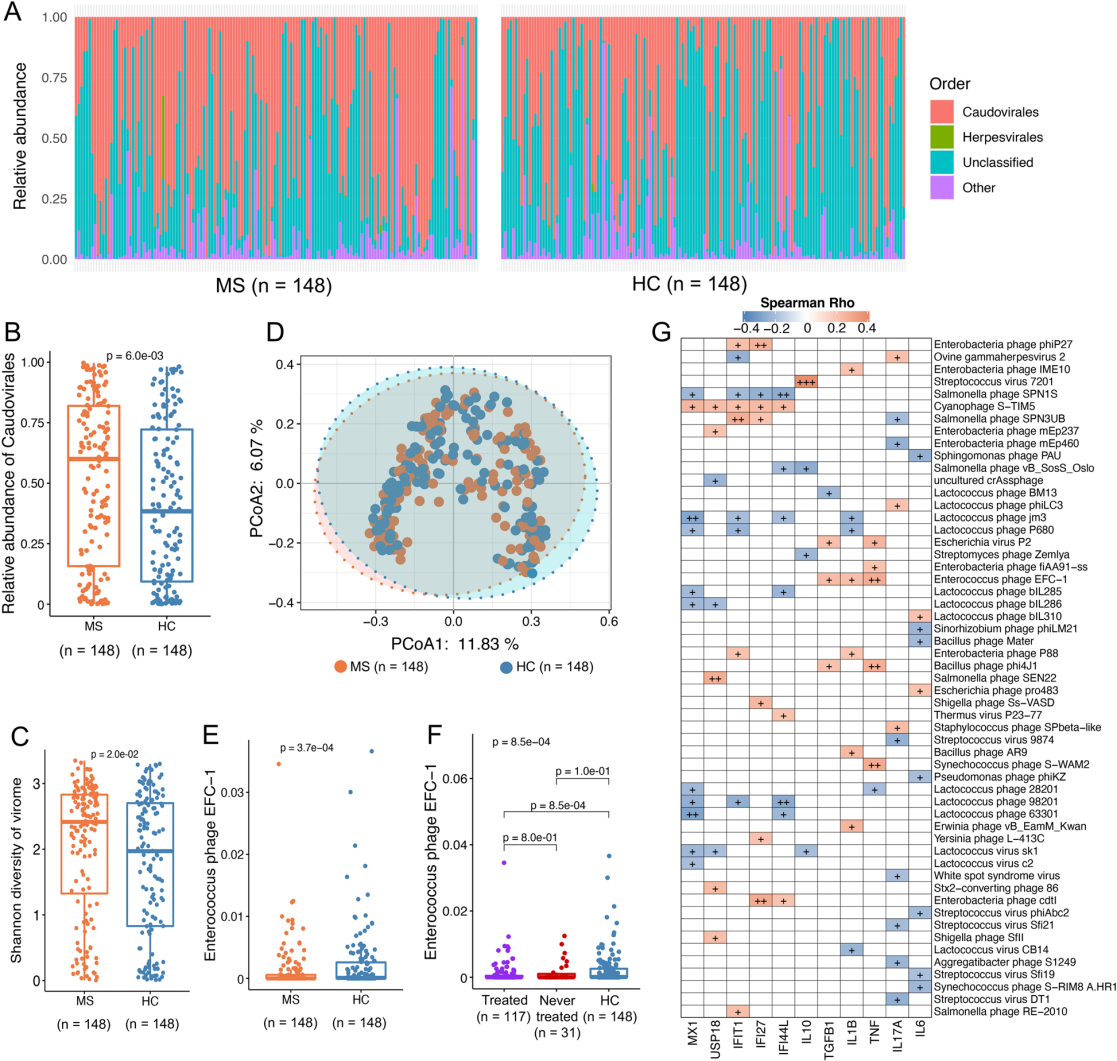
Alteration of viral gut microbiota composition in cases and HC subjects. **A** Relative abundance of gut viral orders in cases and HC groups. **B** Relative abundance of *Caudovirales* in cases and HC individuals. **C** Shannon’s diversity for the viral gut microbiota between patients and HC at the virus species level. Statistical significance was determined by Wilcoxon’s rank sum test between two groups. **D** Principal coordinate analysis (PCoA) of the Canberra distance showing the stratification of patients from HC by viral gut microbiota at species level. Statistical significance for the Canberra distance was determined by PERMANOVA with permutations done 999 times. **E** Relative abundance of bacteriophage *Enterococcus* phage EFC-1 in all cases and HC. **F** Relative abundance of bacteriophage *Enterococcus* phage EFC-1 in treated or never treated cases compared with HC. **G** Gut viral species associate with blood cell expression of inflammation markers. Statistical significance was determined by Wilcoxon’s rank sum test between two groups. Kruskal-Wallis test, followed by Wilcoxon’s rank sum test with Benjamini-Hochberg correction was performed between the three groups. HC = healthy controls

Among the gut viral orders, *Caudovirales* bacteriophages dominated the viral gut microbiota in both cases and HC (Fig. 5A and Additional file 2: Fig. S16A). The same viral order significantly differed between the two groups (Fig. 5B) and was also associated with treatment (Additional file 2: Fig. S16B). To explore a potential aberration of the viral gut microbiota in cases versus HC, we tested the viral alpha diversity indices. We found a significantly higher Shannon diversity of viral species (*P* = 0.022, Fig. 5C) and slightly lower viral Chao1 richness (*P* = 0.058, Additional file 2: Fig. S16C) in cases compared to HC. In addition, we examined the beta diversity of gut viral species by principal coordinates analysis based on the Bray-Curtis distance between individual viromes of the dataset. We found that the composition of the viral microbiota of cases and HC grouped into two clusters (PERMANOVA, *P* = 0.017), suggesting the composition of viral gut microbiota of cases differs from that in HC (Fig. 5D).

In the structure of the viral gut microbiota of treatment-based subgroups of cases, we found a modest change in viral richness of the treatment-naïve cases subgroup when compared with HC (adjusted *P* = 0.056, Additional file 2: Fig. S16D), whereas there was no significant difference in alpha or beta diversity between these subgroups (Additional file 2: Fig. S16E-F). When compared with findings from the bacterial gut microbiota analyses, this finding may suggest that the viral gut microbiota is less responsive to multiple sclerosis treatment than the bacterial microbiota. Additionally, in the subgroup comparisons between CA, CNA, and HC, we found that gut viral composition of CNA individuals differs from that of HC group (adjusted *P* = 0.009, Additional file 1: Table S7).

Only one viral gut microbiota feature differed between cases and HC (Fig. 5E and Additional file 1: Table S8). This feature is annotated as *Enterococcus* phage EFC-1, the abundance of which was lower in cases and inversely associated with drug treatment (Fig. 5F) but positively linked to the blood cell gene expression of pro-inflammatory markers including *IL1B* and *TNF-α* (Fig. 5G). This bacteriophage is reported as a lytic or temperate phage to its bacterial host, *Enterococcus faecalis*, with a prevalence lower than 10% in our dataset (Additional file 2: Fig. S16G). Of interest, multiple *Lactococcus* viruses were inversely linked to inflammatory markers expressed in blood cells (Fig. 5G). Another bacteriophage, *Enterobacteria* phage cdtI, was positively associated with multiple sclerosis treatment, whereas the abundance of its host bacterium *Escherichia coli* was not altered in treated cases (Additional file 2: Fig. S16H-I).

## Discussion

Following full adjustment for inter-individual differences in age, sex, BMI, bacterial cell counts, and various treatment regimens, we found 61 bacterial species differentially abundant when comparing all cases with HC, among which 31 species were increased in multiple sclerosis cases (referred to as MS-related species). More of these have previously been reported to be increased in relative abundance in multiple sclerosis, notably *Clostridium leptum*, *Clostridium inocuum*, *Anaerotruncus colihominis*, or *Ruminococcus gnavus*. However, the majority of the here identified MS-related species have not previously been linked to multiple sclerosis. One of them, *Flavonifractor plautii*, is of particular interest since several studies showed that it may affect IL-17 or CD4+T cells [55, 56]. As an isoflavone-metabolizing species, this increase might be due to an isoflavone-enriched diet in MS cases [57], which could be tested in an interventional clinical trial. Overall, multiple sclerosis-related species were inversely associated with bacterial species richness, while HC-related species were positively associated with richness, suggesting that processes that reduce species richness are linked to the multiple sclerosis gut microbiota. To reinforce this hypothesis, multiple sclerosis-related species did directly associate with a group of biomarkers of inflammation (plasma concentrations of *IL17A*, *IL6*, *USP18*, CRP, and blood counts of total leukocytes, monocytes, neutrophils, and basophils) in the sub-cohort of treatment-naïve cases.

At the level of the bacteriome, we found several pathways related to methane metabolism enriched in cases. This is consistent with other studies, which reported an increase in relative abundance of *Methanobrevibacter smithii* or methane in multiple sclerosis patients [58]. However, this difference might be an effect of constipation in multiple sclerosis. Indeed, *M. smithii* is known to associate with lower transit time. Consistently, some methane metabolism pathways were in fact confounded by fecal water content, a proxy of constipation, in our study.

In treatment-naïve cases, we found a strong and positive relationship between the number of relapses during 2 years of follow-up and bacterial richness, meaning that cases with clinical disease activity (CA) were richer in gut bacteria than clinically not active patients (CNA), a pattern that did not exist in other treatment-defined subgroups of cases. This result is unexpected since a high gut bacterial richness is commonly considered as a beneficial marker of health [36], even if it was reportedly increased in schizophrenia [59]. A longitudinal study should allow following dynamics of gut microbiota and thus determine possible richness changes in remission and relapses. Our observations suggest that richness might be a variable to consider in the context of specific disease and might not generally indicate the disease severity.

The abundance of two bacterial species were enriched in the group of untreated CNA, *Faecalibacterium prausnitzii* and *Gordonibacter urolithinfaciens*. The former is a butyrate-producer well known for its anti-inflammatory properties. The latter produces urolithin, a metabolite that also holds anti-inflammatory properties which alleviates severity of EAE in mice [60, 61]. Both bacterial species were also more abundant in the whole multiple sclerosis group of CNA cases as compared to CA or HC. Whether this finding reflects changes in lifestyle and corresponding changes in abundance of a selected gut bacterial species in CNA cases, for example following adoption of a diet enriched in plant-based phytochemicals including the polyphenol ellagic acid, the precursor of urolithin, is unknown.

Intriguingly, the established properties of these two bacterial species make *F. prausnitzii* and *G. urolithinfaciens* relevant live biopharmaceutical product candidates to be tested in future clinical trials aiming to alleviate multiple sclerosis by decreasing the number of relapses. Our findings are in line with a recent study showing that a diet enriched in isoflavones, another type of polyphenol, alleviates EAE in mice [57].

On the contrary, it is also noteworthy that we identified a positive correlation between several species of the CA-associated bacterial microbiota and plasma concentrations of IL-17A, as well as type 17-linked IL-22. Besides their linkage to active disease, both cytokines also correlate with active brain lesions in multiple sclerosis [62], hence supporting the notion of a gut bacteria-cytokine-brain axis in multiple sclerosis with possible involvement of the identified bacteria.

At the gut bacteriome level, methanogenesis capacity was lower in CNA, which is consistent with a study showing a direct association between the presence of an Euryarchaeota and a shorter time to relapse [63]. However, considering the whole patients cohort and following deconfounding for covariates and adjustment for differences in fecal water content, most significant links between methanogenesis pathways of the gut microbiome and multiple sclerosis were lost. Thus, the reduced methanogenesis potential in multiple sclerosis might be secondary to obstipation, which is a common complication in multiple sclerosis [64]. Indeed, constipation was associated with an altered gut microbiota and worsening of disease in the EAE mouse model [65].

In the whole multiple sclerosis cohort, we also found that propionate metabolism was different between CNA and CA following deconfounding of microbiome data. Serum concentration of propionic acid is reported to be lower in cases compared to HC in several studies [66], and supplementation of propionate has been shown to alleviate multiple sclerosis symptoms [22].

While these three immune-modulating gut bacterial metabolites, butyrate, urolithin, and propionate, may play a crucial role in prevention of relapses of multiple sclerosis, other recent studies have indicated that additional metabolites in blood and cerebrospinal fluid derived from gut bacterial modification of food components may exert neurotoxic effects in multiple sclerosis [24, 67]. Thus, a complex mixture of immune-modulating gut bacterial metabolites triggering disease escalation or de-escalation may be involved in multiple sclerosis pathogenesis. A logical next step in exploring potential causal roles of a disrupted bacterial gut microbiota in multiple sclerosis pathogenesis might therefore be performance of clinically controlled trials combining medical treatment with an adjuvant lifestyle intervention focusing on a predominantly plant-based diet tailored for favoring gut bacterial production of butyrate, propionate, and urolithin. Alternatively, *F. prausnitzii* and *G. urolithinfaciens* or their derived immune-modulating compounds could be devised as probiotics or postbiotics.

The outcome of our studies of the viral gut microbiota is to be considered preliminary since the analyzed metagenomics sequencing reads originated from bulk and not from virus-enriched fecal DNA. However, the findings suggest that the viral microbiota of cases may differ from that of HC. Especially, the finding of a depletion of the *Enterococcus* phage EFC-1 in cases is of interest. The gut bacterial host of this phage, *Enterococcus faecalis*, is well known as an opportunistic pathogen, which may cause severe infections. Therefore, an enrichment of bacteriophage *Enterococcus* phage EFC-1 might be considered a potential target for future explorative intervention in multiple sclerosis.

Limitations of our study include lack of fecal and plasma metabolomics to directly measure potential differences in bacterial metabolites (especially butyrate, propionate, and urolithin) in CA and CNA patients. Gut mycobiome data would also have been helpful in getting the full picture of gut microbiota in MS, as a recent study found gut mycobiome altered in patients with MS [68].

## Conclusions

We demonstrate an aberrant bacterial and viral gut microbiota in multiple sclerosis and that an IL-17A-linked bacterial gut microbiota increases with disease activity. Our studies of non-disease-active cases identify two anti-inflammatory bacterial species, *Faecalibacterium prausnitzii* and *Gordonibacter urolithinfaciens* whose metabolites, butyrate, and urolithin, are known to counteract immune disruption in animal models of multiple sclerosis. These bacterial species or their derived immune-modulating postbiotics are candidates to be tested in future clinically controlled interventions as a microbiota-based adjunct therapy. Alternatively, medical treatment could be combined with a tailored plant-based diet favoring specific gut bacterial production of the identified immune-modulating compounds.

## Supplementary Information

**Additional file 1: Table S1.** Contrasts in plasma cytokines between patients with multiple sclerosis and HC; **Table S2.** Fecal water measurements and bacterial cell count index; **Table S3.** Characteristics of patients at baseline and after follow-up; **Table S4.** Pairwise PERMANOVA between subgroups of patients and HC (tested on Bray-Curtis dissimilarity based on MGS abundance); **Table S5.** Contrasts in Metagenomics Species (MGS) between patients with multiple sclerosis and HC; **Table S6.** Contrast in features related to methanogenesis between CA and CNA in treatment-naïve patients (n = 31); **Table S7.** Pairwise PERMANOVA between subgroups of patients and HC (tested on Bray-Curtis dissimilarity based on viral abundance); **Table S8.** Differential gut viral species between cases and healthy controls identified by MaAslin2 package.

**Additional file 2: Fig. S1.** Flow Cytometry fixed gating strategy; **Fig. S2.** Contrasted cytokines levels; **Fig. S3.** α- and β-diversity of bacterial species; **Fig. S4.** α- and β-diversity of bacterial species and smoking status; **Fig. S5.** α- and β-diversity of bacterial species and treatment; **Fig. S6.** Associations between contrasted bacterial species (metagenomics species (MGS)) and species richness; **Fig. S7.** Associations of contrasted bacterial species (metagenomics species (MGS) with inflammatory markers; **Fig. S8.** Richness of bacterial species (metagenomics species (MGS)) and disease activity in treatment-based subgroups of patients with multiple sclerosis; **Fig. S9.** Bacterial species (metagenomics species (MGS)) richness and relapse delay; **Fig. S10.** Abundance of Flavonifractor plautii; **Fig. S11.** Relation between methanogenesis features and fecal water content; **Fig. S12.** Associations between CA/CNA contrasted bacterial species (metagenomics species (MGS)) and species richness; **Fig. S13.** Correlation between CA/CNA contrasted bacterial species and fasting circulating inflammation markers in HC; **Fig. S14.** Contrasts of bacterial species (metagenomics species (MGS)) between CA and CNA patients with multiple sclerosis; **Fig. S15.** Contrasts of predicted bacterial functional modules between CA and CNA patients with multiple sclerosis; **Fig. S16.** Alterations of gut virome composition in patients with multiple sclerosis and HC.

## Data Availability

Sequencing reads filtered for low quality and human contamination are available from ENA: PRJEB51635 (https://www.ebi.ac.uk/ena/browser/view/PRJEB51635) [69] for multiple sclerosis cases, PRJEB41786 (https://www.ebi.ac.uk/ena/browser/view/PRJEB41786) [70] for the CBMR study and PRJEB41787 (https://www.ebi.ac.uk/ena/browser/view/PRJEB41787) [71] for the DanFunD study. Genes sequences of the IGC2 as well as MGS definition and taxonomy are available from Data INRAe (10.15454/FLANUP) [38].

## Abbreviations

BMI: Body mass index
CA: Clinically active patients
CCL2: Chemokine ligand 2
CD: Cliff’s Delta
CIS: Clinically isolated syndrome
CNA: Clinically not active patients
CRP: C-reactive protein
EAE: Experimental autoimmune encephalomyelitis
EDSS: Expanded Disability Status Scale
GBM: Gut-brain modules
GMM: Gut metabolic modules
HC: Healthy controls
IFN-β: Type-I interferon beta
IGC2: Integrated Gut Catalogue 2
IHMS: International Human Microbiome Standards
IL: Interleukin
KEGG: Kyoto Encyclopedia of Genes and Genomes
KO: KEGG orthologs
MGS: Metagenomic species
MSSS: Multiple Sclerosis Severity Score
NEDA: No evidence of disease activity
PCoA: Principal coordinates analysis
RRMS: Relapsing-remitting multiple sclerosis
SCFAs: Short-chain fatty acids
TGF-β1: Transforming growth factor beta
TNF-α: Tumor necrosis factor alpha
TSLP: Thymic stromal lymphopoietin
