# Supplementary material for: The gut microbiota in multiple sclerosis varies with disease activity
Source: Genome Med. 2023 Jan 5;15:1. doi: 10.1186/s13073-022-01148-1 (PMC9814178; doi:10.1186/s13073-022-01148-1)
Supplement: Supplementary file 2 — Additional file 2: Fig. S1. Flow Cytometry fixed gating strategy; Fig. S2. Contrasted cytokines levels; Fig. S3. α- and β-diversity of bacterial species; Fig. S4. α- and β-diversity of bacterial species and smoking status; Fig. S5. α- and β-diversity of bacterial species and treatment; Fig. S6. Associations between contrasted bacterial species (metagenomics species (MGS)) and species richness; Fig. S7. Associations of contrasted bacterial species (metagenomics species (MGS) with inflammatory markers; Fig. S8. Richness of bacterial species (metagenomics species (MGS)) and disease activity in treatment-based subgroups of patients with multiple sclerosis; Fig. S9. Bacterial species (metagenomics species (MGS)) richness and relapse delay; Fig. S10. Abundance of Flavonifractor plautii; Fig. S11. Relation between methanogenesis features and fecal water content; Fig. S12. Associations between CA/CNA contrasted bacterial species (metagenomics species (MGS)) and species richness; Fig. S13. Correlation between CA/CNA contrasted bacterial species and fasting circulating inflammation markers in HC; Fig. S14. Contrasts of bacterial species (metagenomics species (MGS)) between CA and CNA patients with multiple sclerosis; Fig. S15. Contrasts of predicted bacterial functional modules between CA and CNA patients with multiple sclerosis; Fig. S16. Alterations of gut virome composition in patients with multiple sclerosis and HC. [file 13073_2022_1148_MOESM2_ESM.docx]

Supplementary Materials for

**The gut microbiota in multiple sclerosis varies with disease activity**

Florence Thirion,^†^ Finn Sellebjerg,^†^ Yong Fan,^†^ Liwei Lyu, Tue H. Hansen, Nicolas Pons, Florence Levenez, Benoit Quinquis, Evelina Stankevic, Helle B. Søndergaard, Thomas M. Dantoft, Casper S. Poulsen, Sofia K. Forslund, Henrik Vestergaard, Torben Hansen, Susanne Brix, Annette Oturai, Per Soelberg Sørensen, Stanislav D. Ehrlich, Oluf Pedersen

Corresponding author: Oluf Pedersen. Email: oluf@sund.ku.dk

**This PDF file includes:**

Fig. S1 to Fig. S16


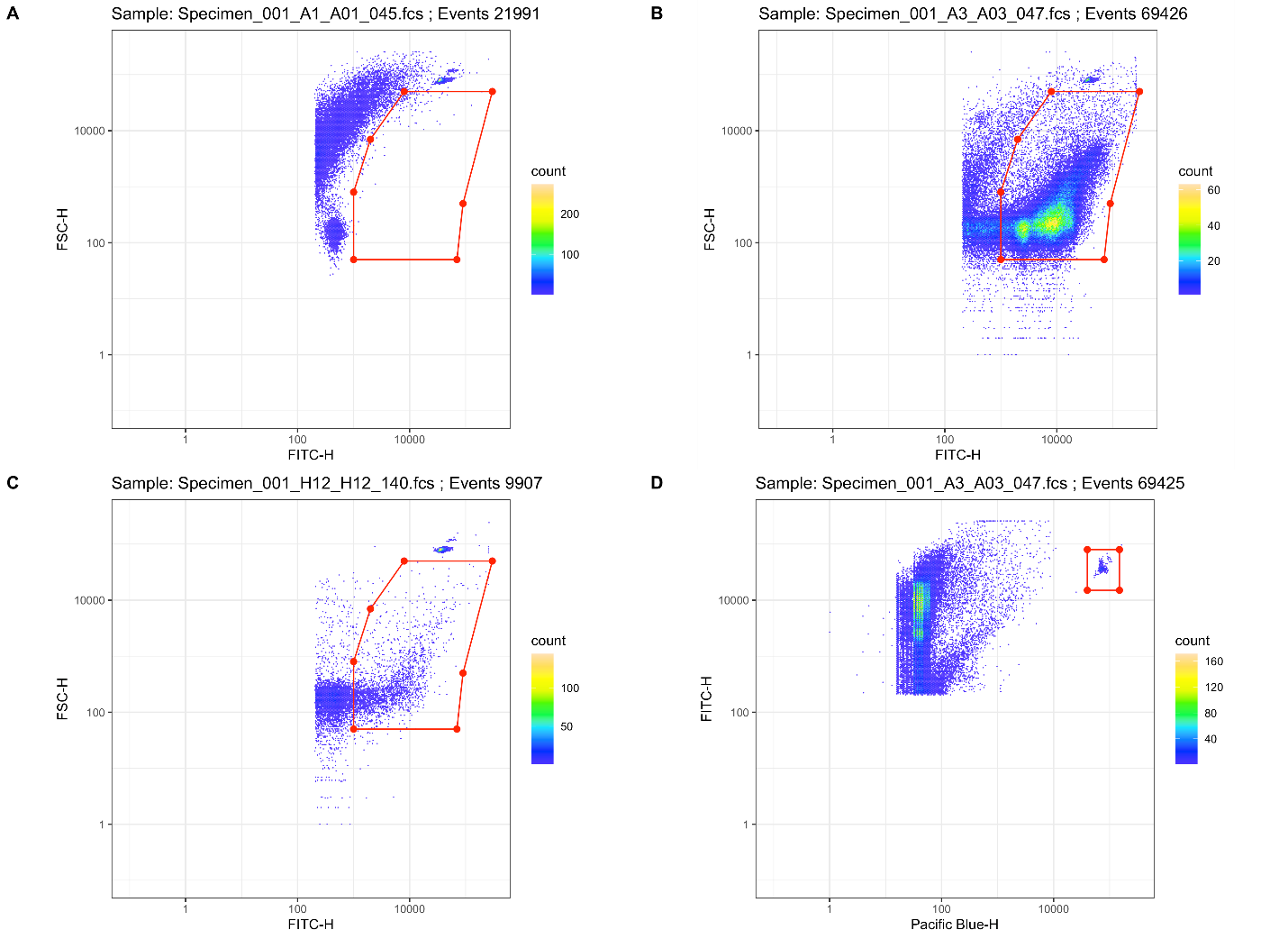


Fig. S1 Flow Cytometry fixed gating strategy. The exact same gating strategy was applied for all fecal samples in the form of a ﬁxed template to allow direct comparison between measured samples. Gating of the bacteria. FITC (530/30 nm) vs. FSC allowed for distinction between the stained bacterial cells and unstained debris. (A) unstained sample; (B) stained sample; (C) staining buffer. Gating of the beads. (D) Pacific Blue (450/50 nm) vs FITC (530/30 nm) allowed for distinction between the counting beads and other particles in the testing solution, including bacteria and unstained debris.


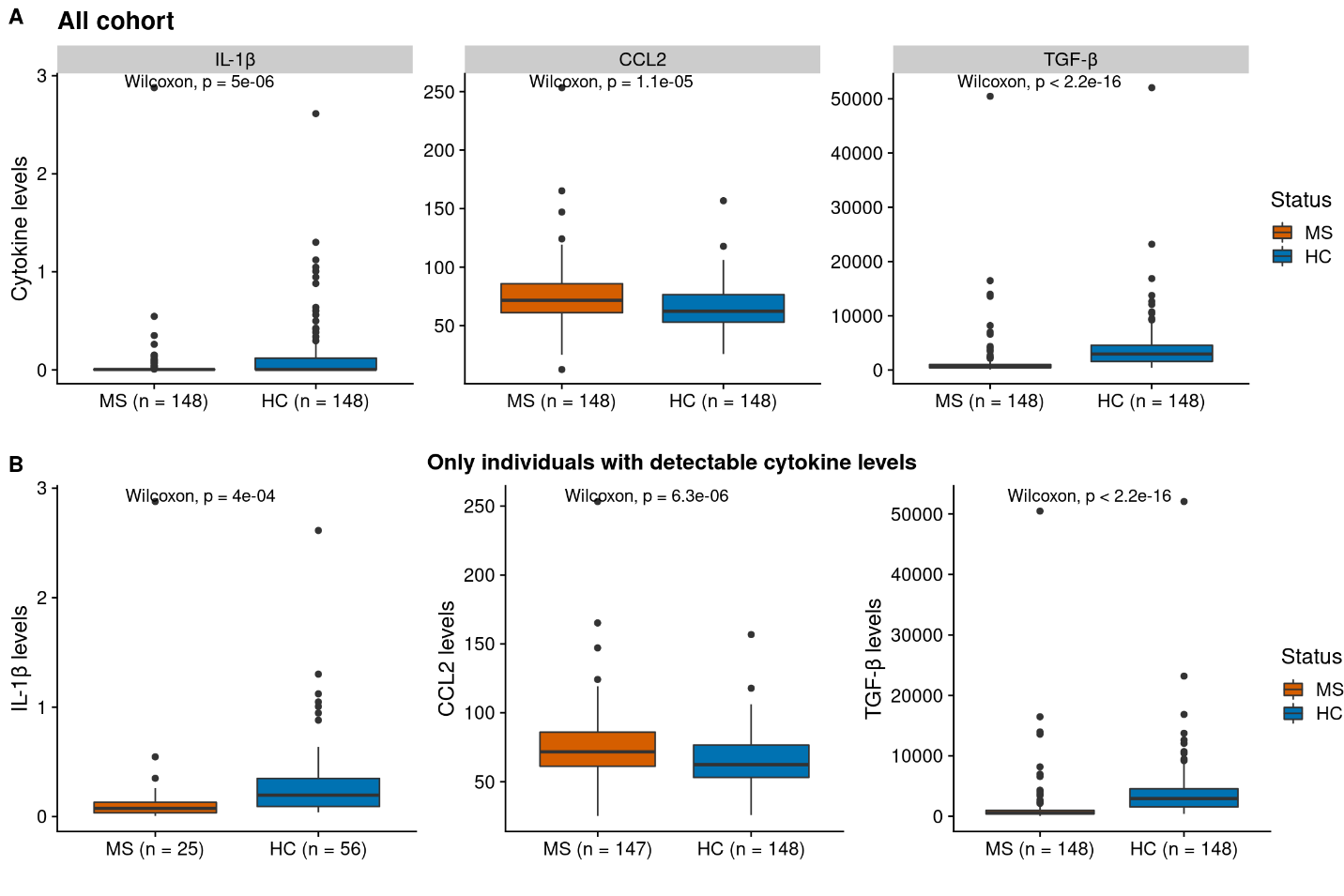


**Fig. S2 Contrasted cytokines levels.** **(A-B)** Distribution of the three contrasted circulating cytokines across patients with MS or HC considering **(A)** all individuals or **(B)** only individuals with detectable cytokine levels. P-values associated with Wilcoxon tests are displayed. *MS = patients with multiple sclerosis, HC = Healthy Controls.*


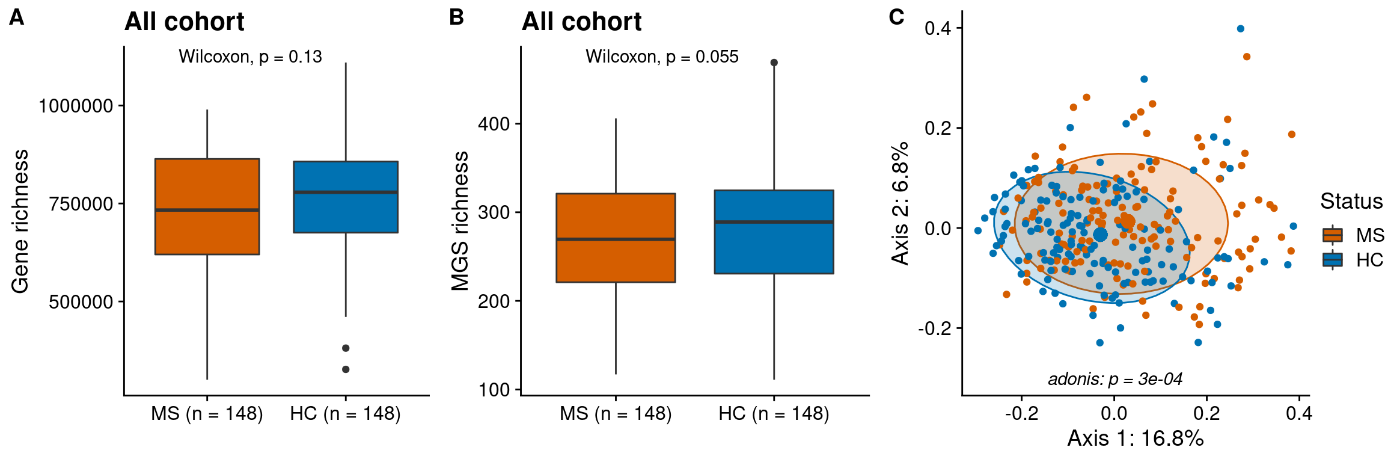


Fig. S3 α- and β-diversity of bacterial species. (A-B) Distribution of genes (A) or species (metagenomics species (MGS)) (B) richness across patients with multiple sclerosis or HC. P-values associated with Wilcoxon tests are displayed. (C) Principal Coordinates Analysis performed on Bray-Curtis dissimilarity matrix computed on MGS abundances. *MS = patients with multiple sclerosis, HC = Healthy Controls, MGS = Metagenomics Species*.


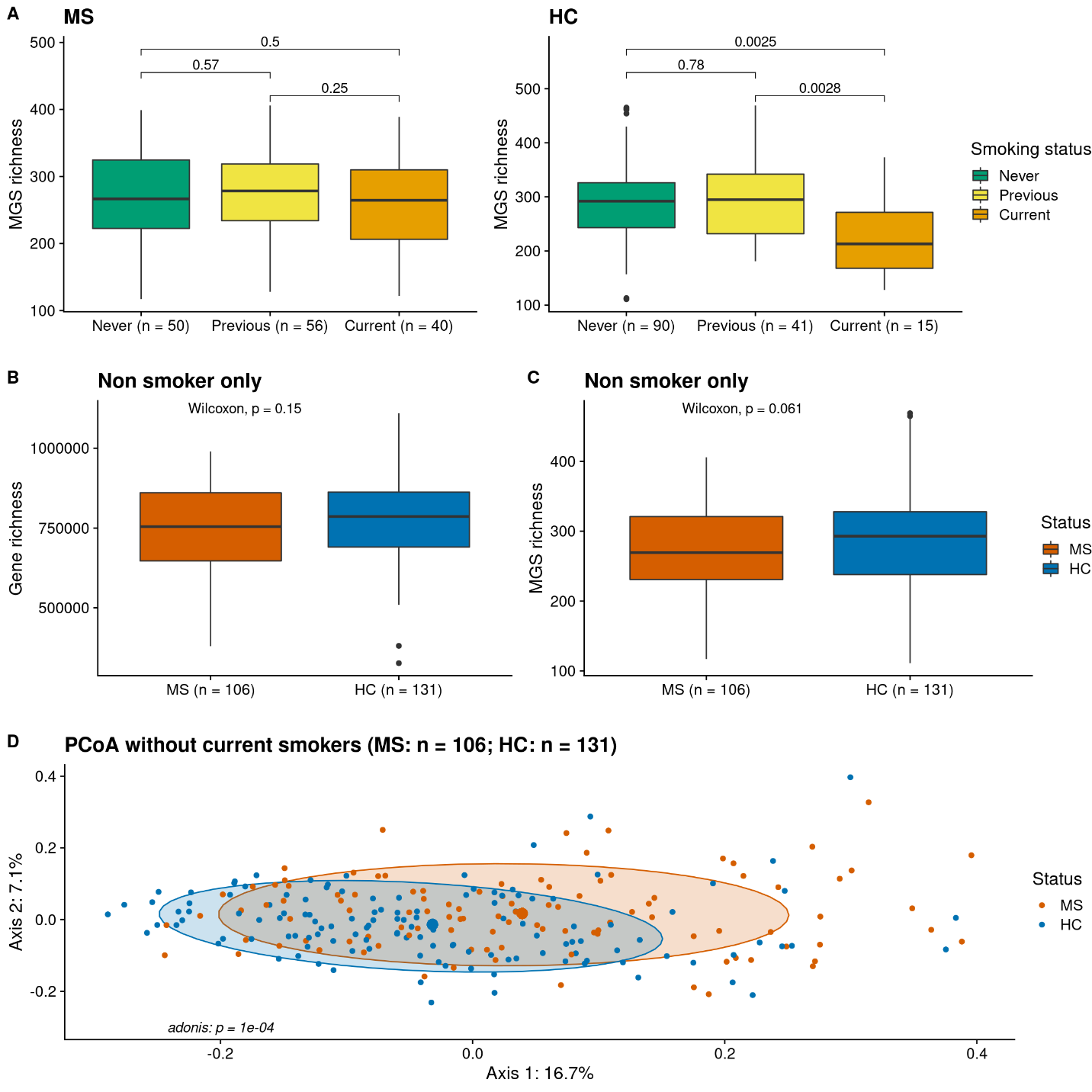


Fig. S4 α- and β-diversity of bacterial species and smoking status. (A) Relation between gene or species (metagenomics species (MGS) richness and smoking status in patients with multiple sclerosis (left) or HC (right). (B-C) Relation between richness and case status when removing current smokers. (D) Principal Coordinates Analysis performed on Bray-Curtis dissimilarity matrix computed on MGS abundances when removing current smokers. *MS = patients with multiple sclerosis; HC = Healthy Controls; MGS = Metagenomics Species.*

**
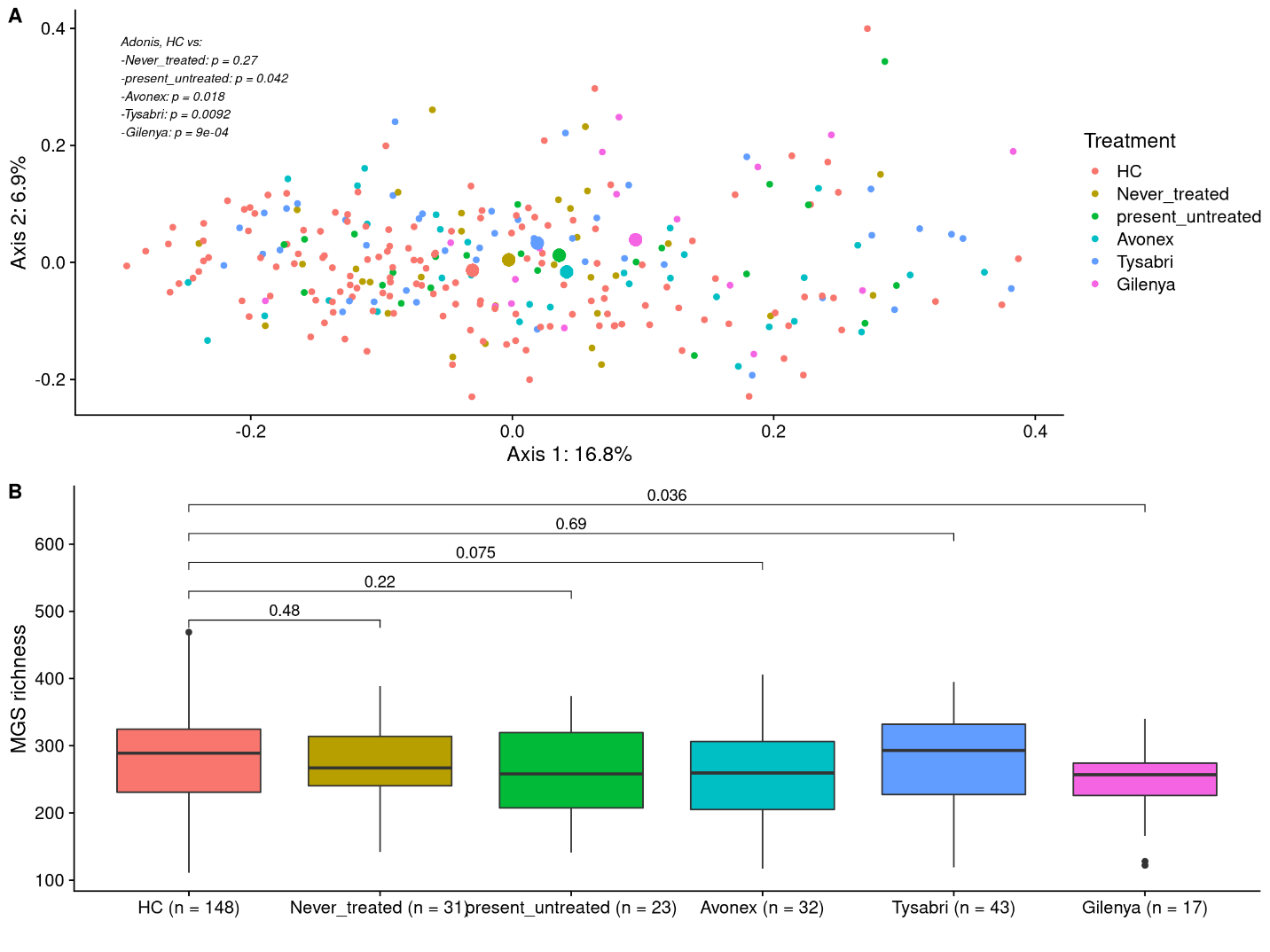
**

**Fig. S5 α- and β-diversity of bacterial species and treatment.** **(A)** Principal Coordinates Analysis performed on Bray-Curtis dissimilarity matrix computed on MGS abundances coloured by treatment. P-values of PERMANOVA performed between HC and each treatment subgroup are displayed. **(B)** Relation between richness and treatment. P-values associated with Wilcoxon tests are displayed. *MGS = Metagenomic Species; HC = Healthy Controls.*


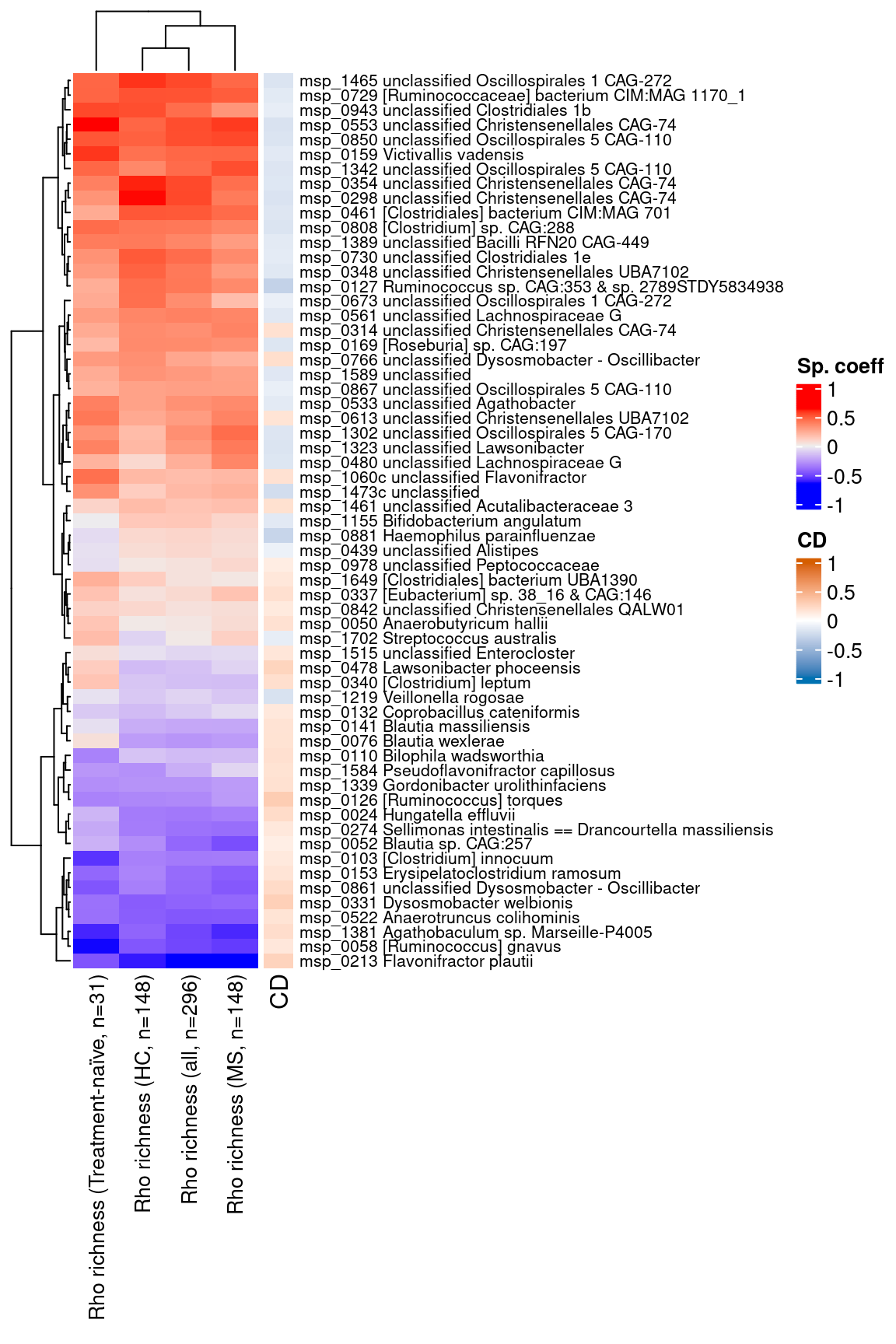


Fig. S6 Associations between contrasted bacterial species (metagenomics species (MGS)) and species richness. Spearman’s correlation coefficient between MGS richness and abundance of contrasted species in different sub-cohorts. The right-side bar indicates Cliff’s Delta (CD, effect size) in the cases/HC contrast (red: more abundant in multiple sclerosis cases; blue: more abundant in HC). *CD = Cliff’s Delta; MS = patients with multiple sclerosis; HC = Healthy Controls; MGS = Metagenomics Species*.


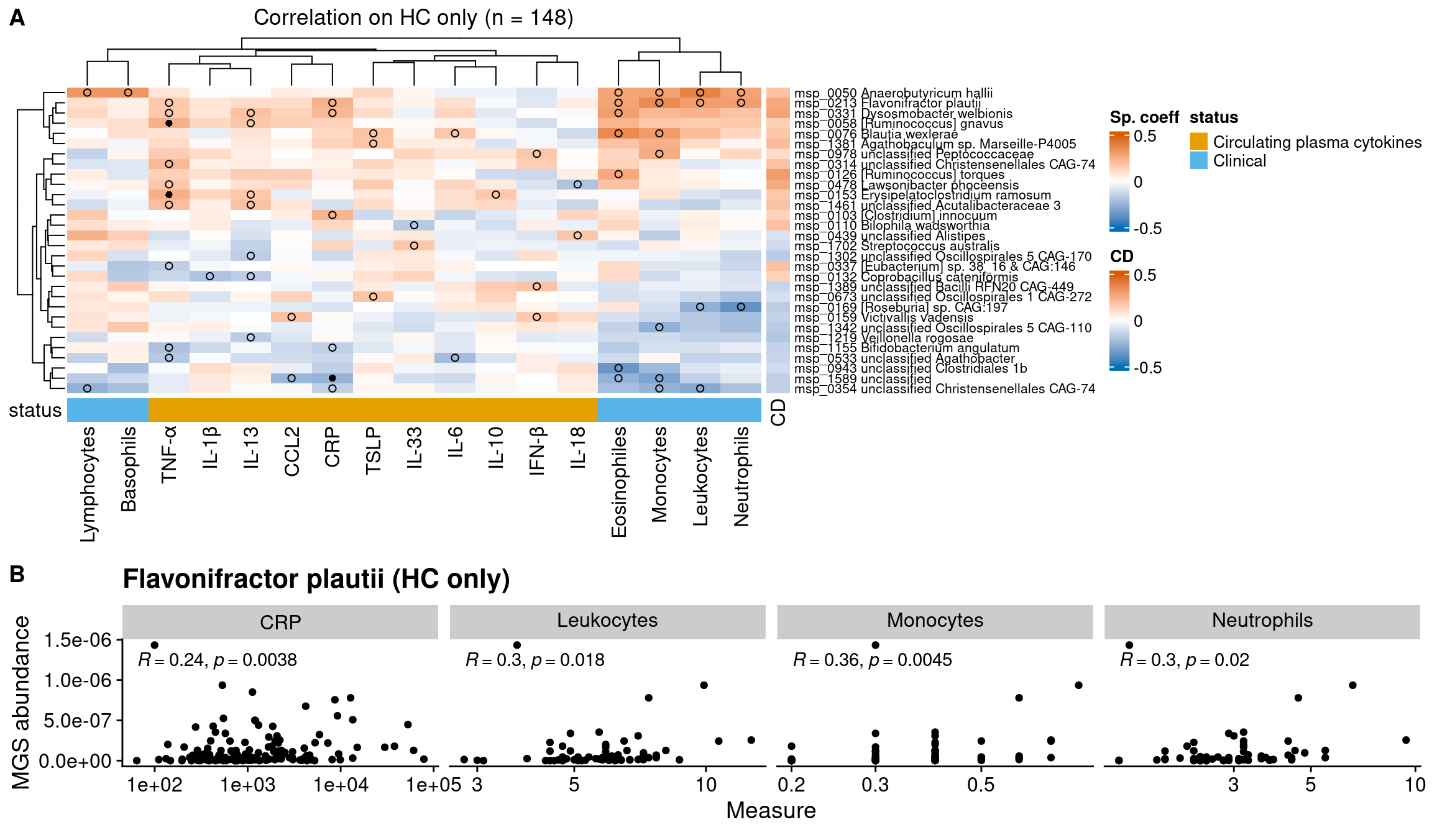


Fig. S7 Associations of contrasted bacterial species (metagenomics species (MGS) with inflammatory markers. (A) Spearman’s correlations between contrasted bacterial species and fasting circulating inflammatory markers in the HC cohort only. Only features with at least one p-value under 0.05 are displayed. Black dots denote correlations with FDR ≤ 0.1, while empty circles indicate correlation with p ≤ 0.05. The right side bars indicate the Cliff’s Delta (CD, effect size) of the feature in the cases/HC contrast (red: more abundant in cases with multiple sclerosis; blue: more abundant in HC). (B) Relationships between abundance of *Flavonifractor plautii* and a group of fasting circulating inflammation markers in the HC cohort only. Spearman’s correlation coefficients along with the associated p-values are displayed. *CD = Cliff’s Delta; MGS = Metagenomics Species, HC = Healthy Controls*.


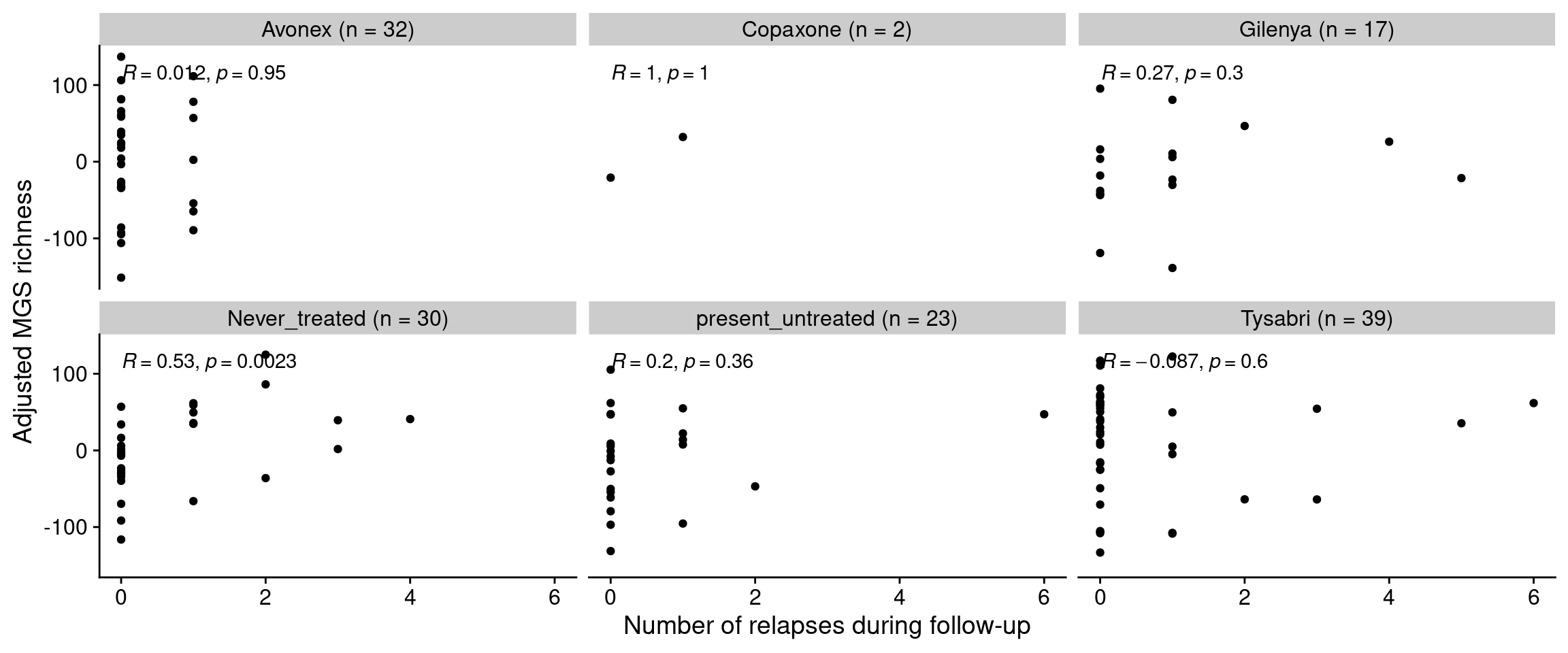


Fig. S8 Richness of bacterial species (metagenomics species (MGS)) and disease activity in treatment-based subgroups of patients with multiple sclerosis. Relationships between species richness adjusted for covariates (age, BMI, smoking status and fecal water content) and number of relapses during follow-up, in different treatment-based subgroups of patients with multiple sclerosis. Spearman’s correlation coefficients along with the associated p-values are displayed. *MGS = Metagenomics Species.*


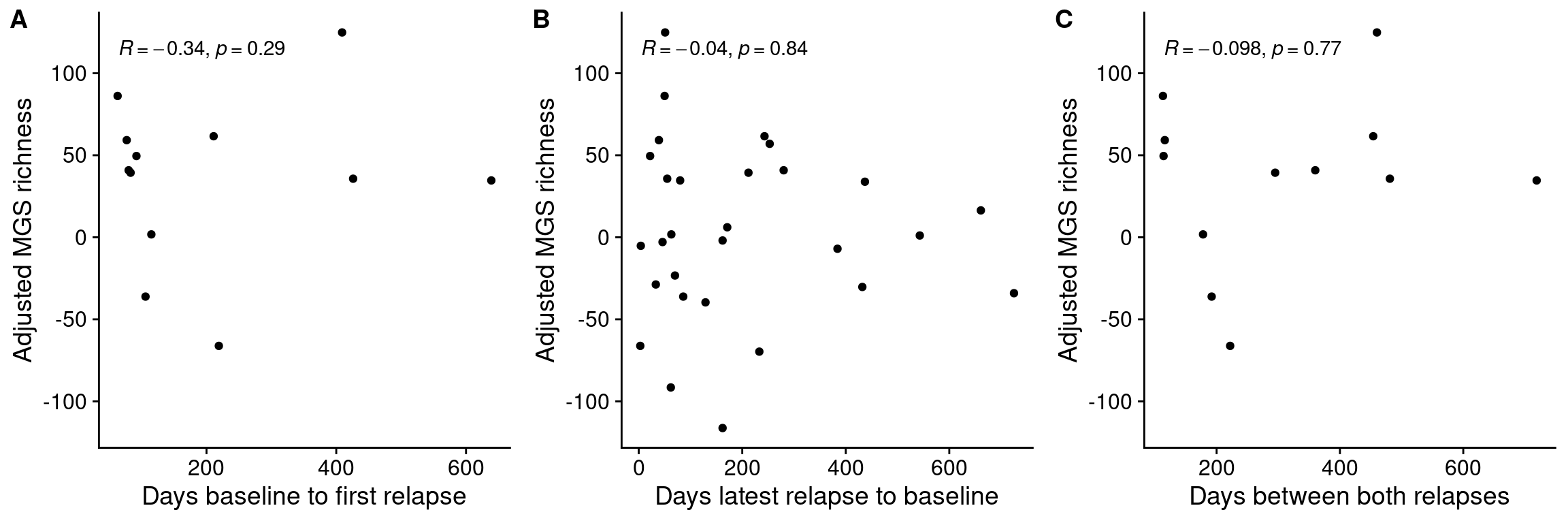


Fig. S9 Bacterial species (metagenomics species (MGS)) richness and relapse delay. Relationships between species richness adjusted for covariates and the time between the latest relapse before baseline and the first relapse after baseline in treatment-naïve patients with multiple sclerosis. Spearman’s correlation coefficients along with the associated p-values are displayed. *MGS = Metagenomics Species.*


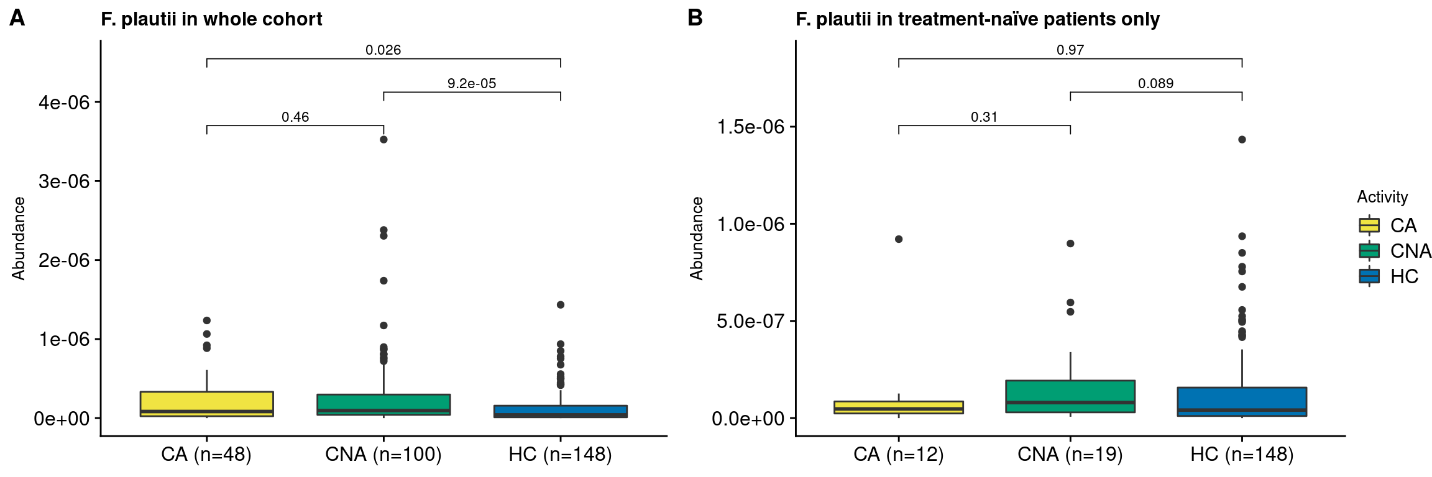


**Fig. S10 Abundance of Flavonifractor plautii**. (A-B) Distribution of F. plautii in CA, CNA and HC considering **(A)** all individuals or **(B)** only HC and treatment-naïve patients. P-values associated with Wilcoxon tests are displayed. *CA = Clinically Active patients; CAN = Clinically Non Active patients; HC = Healthy Controls.*


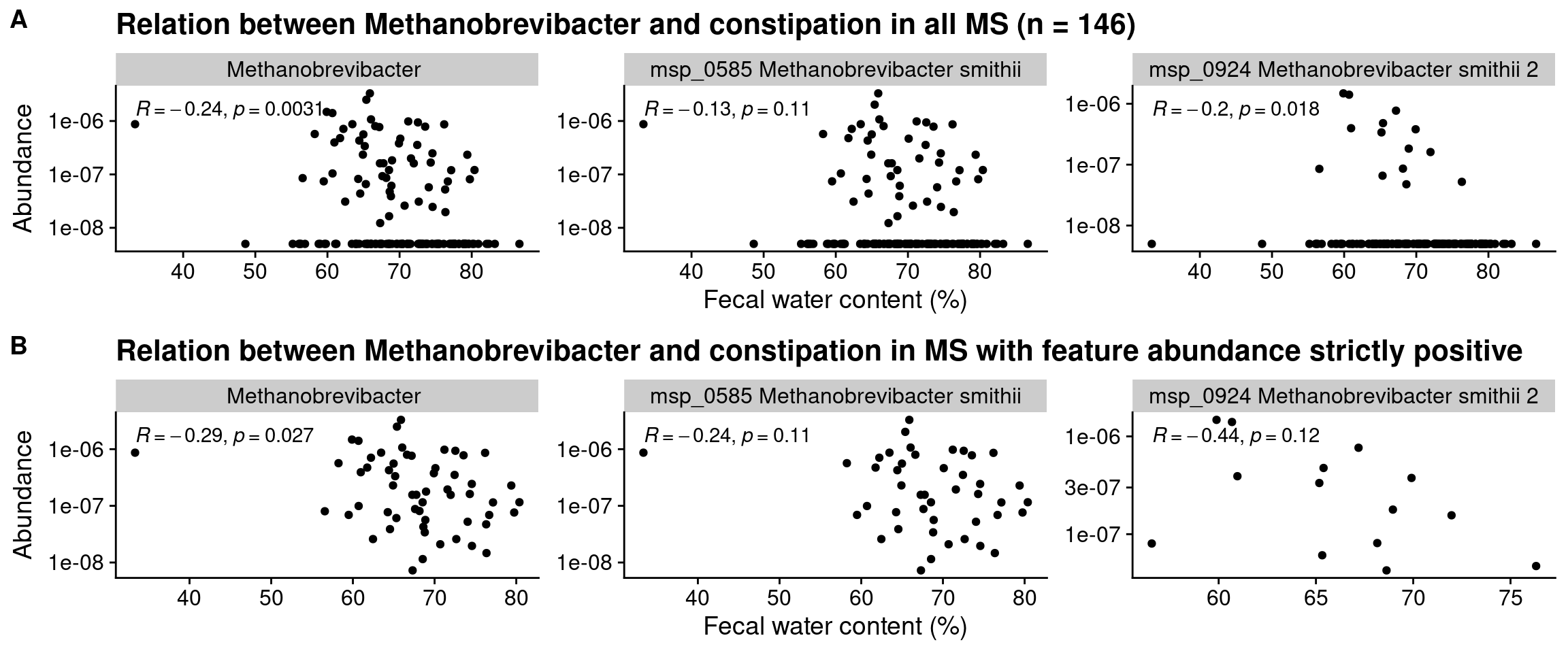


**Fig. S11 Relation between methanogenesis features and fecal water content.** **(A-B)** Scatter plots of three methanogenesis features (the genus *Methanobrevibacter* and two MGS annotated as *Methanobrevibacter smithii*) and fecal water content (proxy of constipation), either in all MS **(A)** or considering only patients whose abundance of a given feature is strictly positive **(B)**. Spearman’s coefficient along with the associated p-value are displayed. *MGS = Metagenomic Species*, *MS = Multiple Sclerosis*.


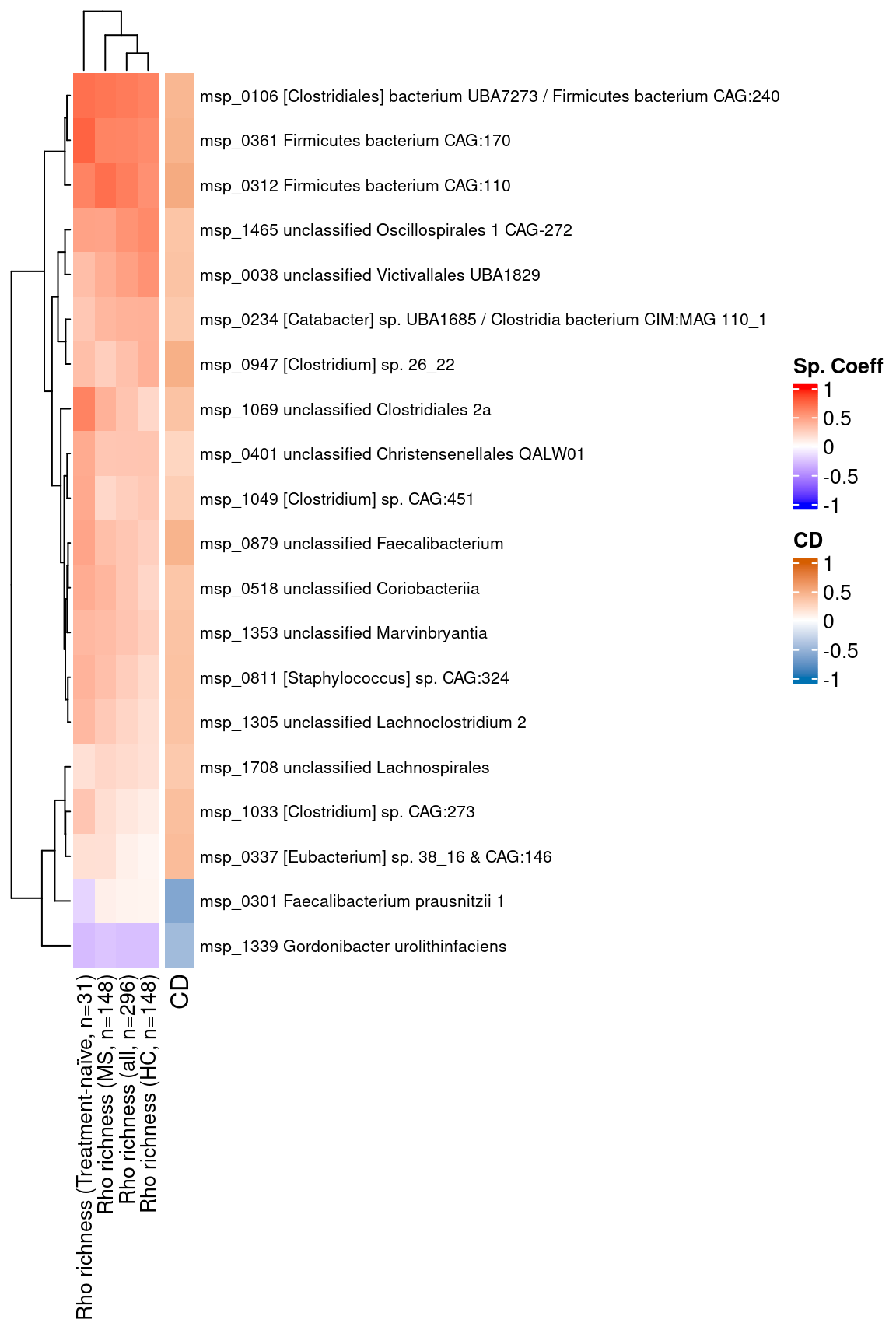


Fig. S12 Associations between CA/CNA contrasted bacterial species (metagenomics species (MGS)) and species richness. Spearman’s correlation coefficient between MGS richness and abundance of CA/CNA contrasted species in different sub-cohorts. The right-side bar indicates Cliff’s Delta (CD, effect size) in the CA/CNA contrast (red: more abundant in CA; blue: more abundant in CNA). *CD = Cliff’s Delta; CA = Clinically Active patients; CNA = Clinically Non Active patients; MGS = Metagenomics Species*.


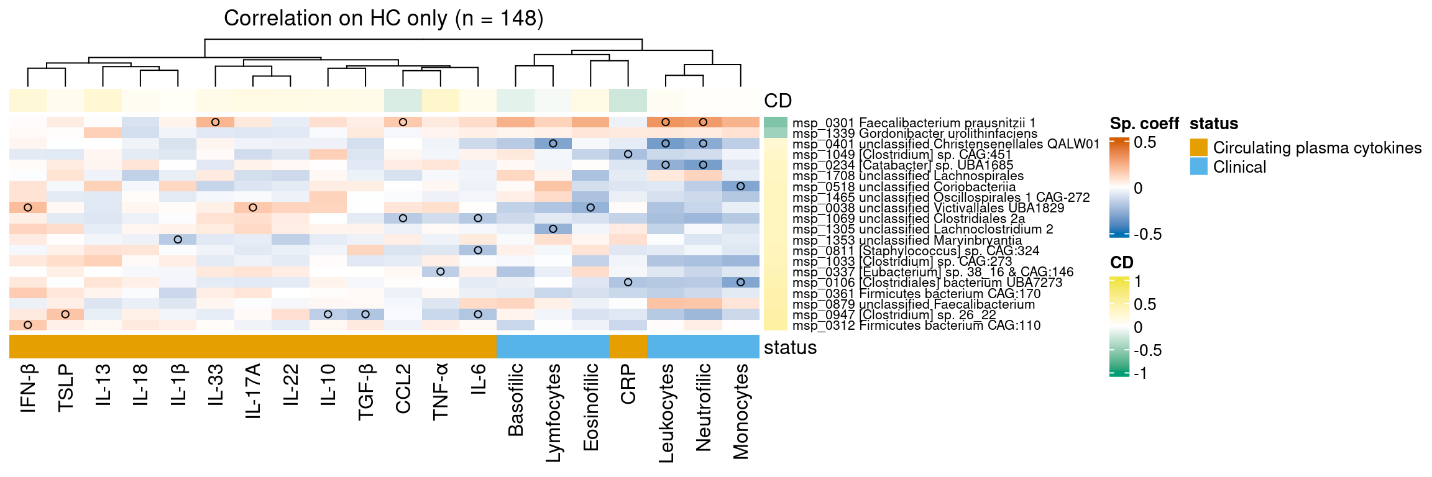


**Fig. S13 Correlation between CA/CNA contrasted bacterial species and fasting circulating inflammation markers in HC.** Only bacterial species with at least one p-value under 0.05 are displayed. Empty circles indicate correlation with *P* ≤ 0.05. The right side bars indicate the Cliff’s Delta (CD, effect size) of the feature in the CA/CNA contrast (green: more abundant in CNA; yellow: more abundant in CA). *CD = Cliff’s Delta; CA = Clinically Active; CNA = Clinically Non Active; HC = Healthy Controls.*


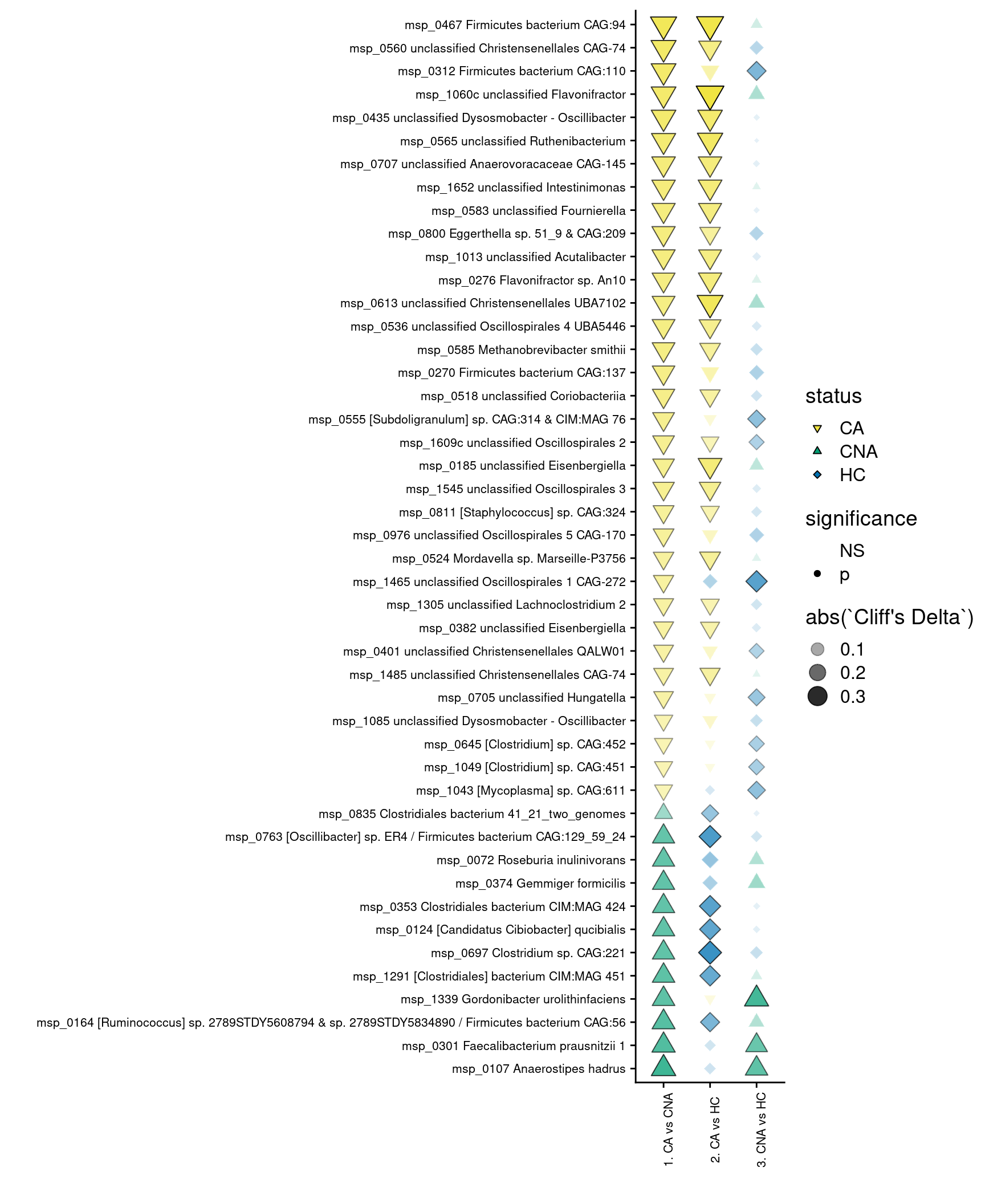


Fig. S14 Contrasts of bacterial species (metagenomics species (MGS)) between CA and CNA patients with multiple sclerosis. Bacterial species that are contrasted between CA and CNA patients (after deconfounding for age, BMI, smoking status, fecal water content and treatment), considering the whole patients cohort. Along is their effect size (Cliff’s Delta) in the contrasts (1) CA vs CNA, (2) CA vs HC, (3) CNA vs HC. *CA = Clinically Active; CNA = Clinically Non Active; HC = Healthy Controls; MGS = Metagenomics Species; NS = Non significant*.


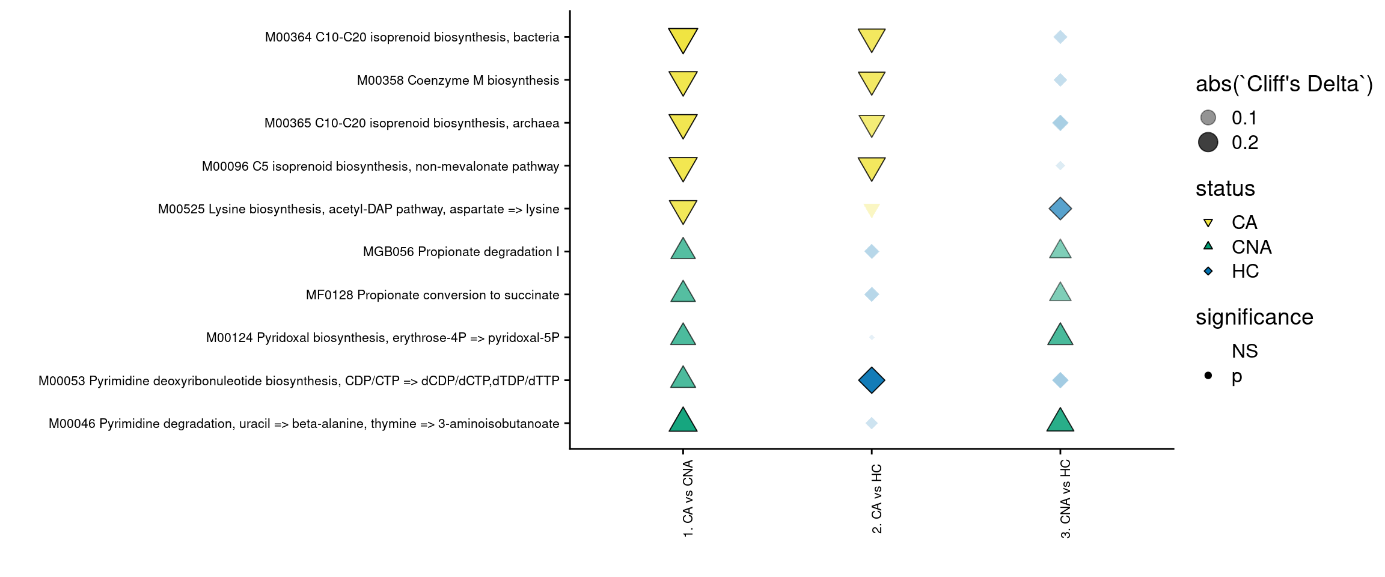


Fig. S15 Contrasts of predicted bacterial functional modules between CA and CNA patients with multiple sclerosis. Predicted functional modules that are contrasted between CA and CNA patients (after deconfounding for age, BMI, smoking status, fecal water content and treatment), considering the whole patients cohort. Along is their effect size (Cliff’s Delta) in the contrasts (1) CA vs CNA, (2) CA vs HC, (3) CNA vs HC. *CA = Clinically Active; CNA = Clinically Non Active; MS = Multiple Sclerosis; HC = Healthy Controls; NS = Non significant.*


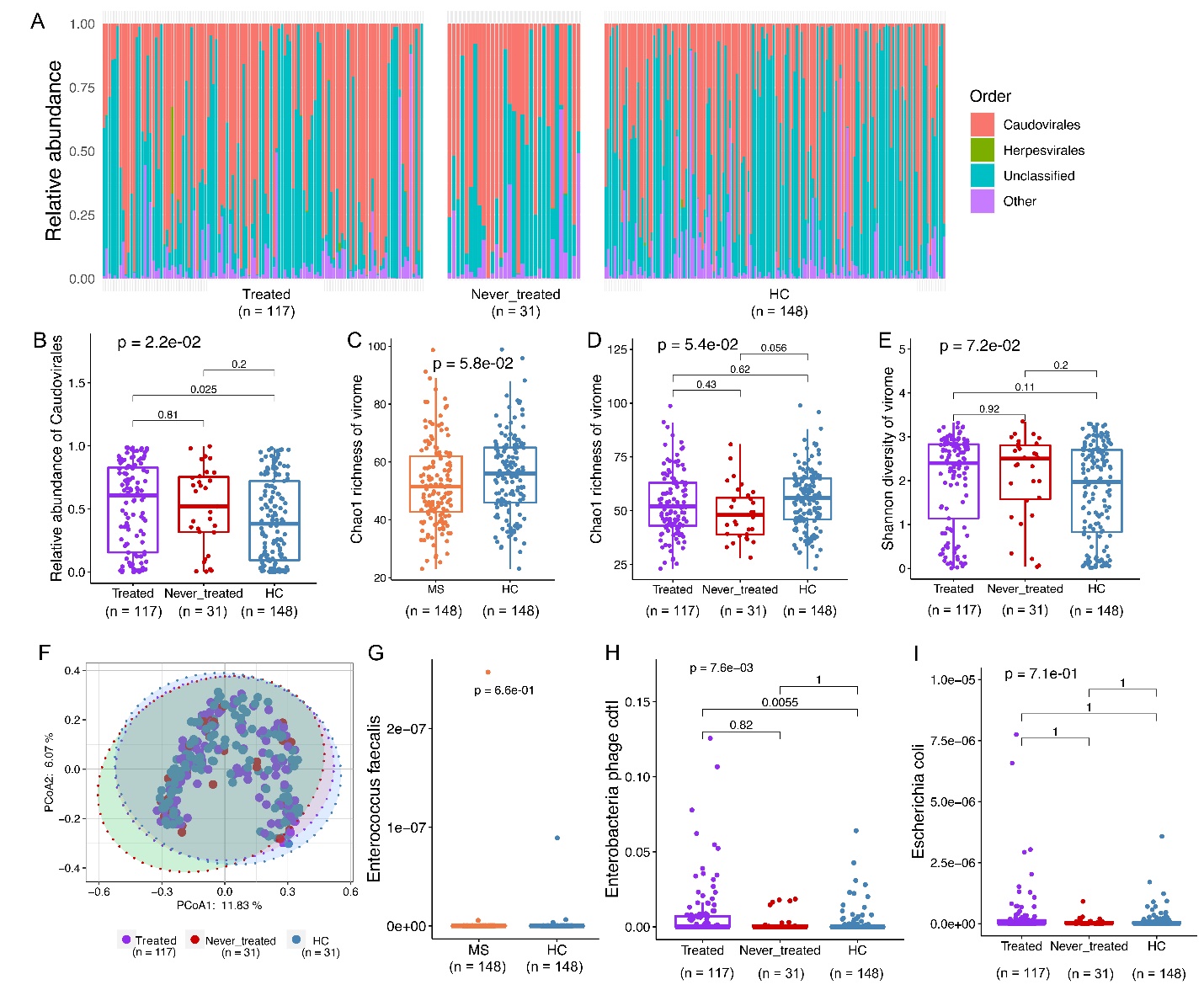


Fig. S16 Alterations of gut virome composition in patients with multiple sclerosis and HC. (A) Relative abundance of gut viral orders in treated or never treated cases and HC individuals. (B) Relative abundance of *Caudovirales* in treated or never treated cases and HC subjects. (C-E) Chao1 richness and Shannon’s diversity for the gut virome between patients and treatment-based subgroups of cases and healthy controls at the species level. Statistical significance was determined by Wilcoxon’s rank sum test between two groups. Kruskal-Wallis test, followed by Wilcoxon’s rank sum test with Bonferroni’s correction was performed for richness and alpha diversity indices between the three groups. (F) Principal coordinate analysis (PCoA) of the Canberra distance showing the stratification of treatment-based MS subgroups from healthy controls by gut virome. Statistical significance for the Bray-Curtis distance was determined by PERMANOVA with permutations done 999 times. (G) Relative abundance of *Enterococcus faecalis* in cases and HC individuals. (H-I) Relative abundance of bacteriophage *Enterobacteria phage* cdtl (H) and its host bacterial species *Escherichia coli* (I) in cases and its treatment-based subgroups. Statistical significance was determined by Kruskal-Wallis test, followed by Wilcoxon’s rank sum test with Bonferroni’s correction was performed between the three groups. *MS = patients with multiple sclerosis; HC = Healthy Controls.*
